# Foxf2 plays a dual role during transforming growth factor beta-induced epithelial to mesenchymal transition by promoting apoptosis yet enabling cell junction dissolution and migration

**DOI:** 10.1186/s13058-018-1043-6

**Published:** 2018-10-01

**Authors:** Nathalie Meyer-Schaller, Chantal Heck, Stefanie Tiede, Mahmut Yilmaz, Gerhard Christofori

**Affiliations:** 1 Department of Biomedicine, University of Basel, Mattenstrasse 28, 4058 Basel, Switzerland; 2 Present address: Institute of Pathology, University Hospital of Basel, Basel, Switzerland; 3 Present address: Integra Biosciences AG, Zizers, Switzerland; 4 Present address: Roche Pharma, Basel, Switzerland

**Keywords:** Apoptosis, Breast cancer, Cell migration, E-cadherin, EGFR, EMT, Foxf2, Noxa

## Abstract

**Background:**

The most life-threatening step during malignant tumor progression is reached when cancer cells leave the primary tumor mass and seed metastasis in distant organs. To infiltrate the surrounding tissue and disseminate throughout the body, single motile tumor cells leave the tumor mass by breaking down cell-cell contacts in a process called epithelial to mesenchymal transition (EMT). An EMT is a complex molecular and cellular program enabling epithelial cells to abandon their differentiated phenotype, including cell-cell adhesion and cell polarity, and to acquire mesenchymal features and invasive properties.

**Methods:**

We employed gene expression profiling and functional experiments to study transcriptional control of transforming growth factor (TGF)β-induced EMT in normal murine mammary gland epithelial (NMuMG) cells.

**Results:**

We identified that expression of the transcription factor forkhead box protein F2 (Foxf2) is upregulated during the EMT process. Although it is not required to gain mesenchymal markers, Foxf2 is essential for the disruption of cell junctions and the downregulation of epithelial markers in NMuMG cells treated with TGFβ. Foxf2 is critical for the downregulation of E-cadherin by promoting the expression of the transcriptional repressors of E-cadherin, Zeb1 and Zeb2, while repressing expression of the epithelial maintenance factor Id2 and miRNA 200 family members. Moreover, Foxf2 is required for TGFβ-mediated apoptosis during EMT by the transcriptional activation of the proapoptotic BH3-only protein Noxa and by the negative regulation of epidermal growth factor receptor (EGFR)-mediated survival signaling through direct repression of its ligands betacellulin and amphiregulin. The dual function of Foxf2 during EMT is underscored by the finding that high Foxf2 expression correlates with good prognosis in patients with early noninvasive stages of breast cancer, but with poor prognosis in advanced breast cancer.

**Conclusions:**

Our data identify the transcription factor Foxf2 as one of the important regulators of EMT, displaying a dual function in promoting tumor cell apoptosis as well as tumor cell migration.

**Electronic supplementary material:**

The online version of this article (10.1186/s13058-018-1043-6) contains supplementary material, which is available to authorized users.

## Background

The process of epithelial to mesenchymal transition (EMT) describes a complex molecular and cellular program by which epithelial cells abandon their differentiated features and acquire mesenchymal characteristics, including motility, invasiveness, and increased resistance to apoptosis [1–5]. EMT has been implicated in several physiological as well as pathological processes. While it is a critical mechanism for embryonic development, EMT is re-engaged in adults during wound healing, tissue regeneration, organ fibrosis, and cancer progression and metastasis [2]. Recent studies implicate that primary tumors displaying an EMT-like gene expression profile are more likely to be associated with distant metastasis formation and a worse prognosis for overall survival [6–8]. In contradiction to these findings are the observations that distant metastases frequently exhibit an epithelial phenotype highly similar to the primary tumor [9, 10]. Explaining this observation, it has been shown that disseminated mesenchymal cancer cells undergo the reverse process (mesenchymal to epithelial transition (MET)) after metastatic spread and colonization and revert to a differentiated, epithelial cell state enabling them to establish in the distant location [11–13]. However, the contribution of EMT to the metastatic process is debated. Recent lineage tracing experiments have suggested that EMT is required for the development of drug resistance but not for metastasis [14, 15]. However, these reports have been met with great skepticism and data questioning these results [16–18]. In summary, overwhelming evidence supports the conclusion that EMT and its reverse process MET are pivotal regulators of cell plasticity in malignant tumor progression and play important roles in drug resistance, relapse, and metastatic progression [19].

Recently, a number of transcription factors have been identified that play critical roles in the initiation and execution of an EMT and in the metastatic process, including Snai1 (Snail), Snai2 (Slug), Zeb1 (δEF1), Zeb2 (Sip1), E47, Twist, goosecoid, Foxc2, Dlx2, RBPjκ, Yap/Taz, Sox4 and 9, Klf4, and NFκB [3, 19–22]. We have previously established a list of genes that change in their expression during the consecutive morphological states of transforming growth factor (TGF)β-induced EMT in normal murine mammary gland (NMuMG) epithelial cells [20, 23]. This analysis identified forkhead box protein F2 (Foxf2) as a transcription factor that is upregulated in its expression during EMT in NMuMG cells and in several other experimental EMT systems. The family of Forkhead box (Fox) genes are defined by a conserved DNA binding domain of a winged helix structure acting as transcription factors, which have been found to serve as key regulators in embryogenesis, signal transduction, maintenance of differentiated cell states, and tumorigenesis [24]. There are three families of Fox genes, Foxc, Foxf, and Foxl1, that form paralogous clusters in the genome and that are extensively expressed in mesodermal tissue [25, 26]. One of the best characterized members of this family is Foxc2, which has been implicated in the regulation of EMT by interacting with Smad proteins and to be a key player in metastasis [27, 28]. Moreover, Foxc1 and Foxc2 are highly expressed in the claudin-low metaplastic breast cancer subtype, which is associated with EMT and cancer stemness [29]. Furthermore, the overexpression of Foxm1 in pancreatic cancer cells leads to the acquisition of an EMT phenotype via upregulation of Zeb1 and Zeb2 as well as stem cell-like characteristics [30].

Foxf2 (also known as Freac-2 or Fkhl6) is a widely expressed protein in various mesenchymal tissues and was first identified as a transcriptional activator containing a forkhead domain for nuclear localization and two independent C-terminal activation domains [31]. Foxf2 interacts with TBP and TFIIB, two components of the general transcriptional activator complex binding a specific DNA motif [32, 33]. Expression of several Fox family genes, including Foxf1 and Foxf2, is specifically regulated by sonic hedgehog (Shh) signaling in a crosstalk with Notch, epidermal growth factor (EGF)/fibroblast growth factor (FGF), and TGFβ signaling [34, 35]. Indeed, TGFβ-induced EMT is one of the mechanisms strongly involved in regulating fusion of the palatal cleft, and Foxf2 levels are high in the mesenchyme of the secondary palate and in the mesenchyme of the lung and gut [36, 37]. Accordingly, Foxf2-deficient mice die shortly after birth due to cleft palate and abnormal tongue and gut development, indicating an essential role of Foxf2 in this EMT-associated developmental process [38, 39]. Epithelial cells of Foxf2-deficient mice show typical signs of depolarization, and the subcellular localization of adherens junctions, normally confined to lateral membranes, expands into the basal and apical membranes.

In many cancer types, the expression of Foxf2 is repressed by promoter hypermethylation or by oncogenic microRNAs (miRNAs), such as miR-301, which promotes breast cancer cell proliferation, invasion, and tumor growth [40, 41], indicating that Foxf2 may act as a tumor suppressor. However, other studies have reported a protumorigenic role of Foxf2 in other cancer types (reviewed in [41]), for example by repressing intestinal stem cells and preventing adenoma formation by inhibiting Wnt signaling [42]. Furthermore, low Foxf2 expression has been reported to correlate with early-onset metastasis and poor prognosis in breast cancer patients [43], and loss of Foxf2 expression promotes an EMT and metastasis of experimental cancer [44, 45]. These conflicting results seem to mirror a double-sided role for Foxf2 in maintaining tissue homeostasis, in regulating an EMT, and in breast cancer progression [46].

Here, we have employed a TGFβ-induced EMT in NMuMG cells and in murine and human breast cancer cells to demonstrate a critical role of Foxf2 during an EMT by concomitantly regulating an EMT, cell survival, and apoptosis. The mechanistic insights into Foxf2 functions also support a dual role of Foxf2 in breast cancer progression and metastasis, on one hand by affecting cell junction homeostasis, and by regulating cell proliferation and survival on the other hand.

## Methods

### Reagents and antibodies

See Additional file 1.

### Cell culture and cell lines

All reagents used for cell culture were obtained from Sigma/Fluka (Basel, Switzerland) if not otherwise mentioned. All cells were cultured at 37 °C with 5% CO_2_ in Dulbecco’s modified Eagle’s medium (DMEM) supplemented with glutamine (2 mM), penicillin (100 U), streptomycin (0.2 mg/l) and 10% fetal bovine serum (FBS). The subclone NMuMG/E9 (hereafter called NMuMG) is expressing E-cadherin and has previously been described [47]. MTΔEcad and MCF7-shEcad have been described previously [23]. NMuMG-shSmad4 and NMuMG-shCtr were obtained from P. ten Dijke (Leiden University Medical Center, The Netherlands) [48]. Py2T breast cancer cells were established from a tumor of the MMTV-PyMT mouse model of breast cancer as previously described [49]. NMuMG cells were treated with TGFβ (2 ng/ml) without serum deprivation, and TGFβ was replenished every 2 days. siRNA transfections with lipofectamine RNAiMAX (Invitrogen) were performed according to the manufacturer’s protocol 24 h before treatment with TGFβ.

### Generation of lentivirus

A cDNA encoding Foxf2 (kindly provided by Leif Lundh, Goteborg University, Sweden) [50] was tagged N-terminally with HA-tag and cloned into the lentiviral expression vector pLenti-CMV-Puro (kindly provided by Matthias Kaeser, Bern). Lentiviral particles were produced by transfecting HEK293T cells with the lentiviral expression vectors in combination with the packaging vector pR8.91 and the envelope encoding vector pVSV using Fugene HD (Roche). After 2 days, the virus-containing HEK293T supernatant was harvested, filtered (0.45 μm), supplemented with polybrene (8 ng/ml), and used for target cell infection. Infections were performed twice a day on 2 consecutive days.

### Growth curves

One day before t_0_, 1.6 × 10^4^ NMuMG cells were seeded in triplicate into 24-well plates and transfected with the indicated siRNA. After 24 h the cells were treated with TGFβ and cell numbers were determined using a Neubauer counting chamber.

### Migration assay

NMuMG cells (2 × 10^4^/well) pretreated for 18 days with TGFβ were seeded in DMEM, 2% FBS, and TGFβ into the upper chamber of a cell culture insert (pore size 8 μm; Falcon BD, Franklin Lakes, NJ). The lower chamber was filled with DMEM, 20% FBS, and TGFβ. After 16 h incubation at 37 °C and 5% CO_2_, the cells that had traversed the membrane were fixed in 4% paraformaldehyde/phosphate-buffered saline (PBS) (15 min at room temperature), stained with DAPI (0.5 μg/ml), and counted using a fluorescence microscope.

### Quantitative real-time polymerase chain reaction (RT-PCR)

Total RNA was prepared using Tri Reagent (Sigma-Aldrich), reverse transcribed with ImProm-II Reverse Transcriptase (Promega) and transcription levels were quantified using SYBR-green PCR Mastermix (Eurogentec) in a real-time PCR system (Step One Plus, Applied Biosystems). Human or mouse riboprotein L19 primers were used for normalization. PCR assays were performed in duplicate and the fold induction was calculated against control-treated cells using the comparative Ct method (ΔΔC_t_). To quantify miRNA levels, RNA was isolated with the miRNeasy kit (Qiagen) followed by polyadenylation and reverse transcription using QuantiMir RT kit (BioCat). Primers are listed in Additional file 1.

### Immunoblotting and immunofluorescence staining

See Additional file 1.

### Apoptosis assay

Cells were washed twice in ice-cold PBS and suspended in 1× Annexin-V binding buffer (0.01 M HEPES, pH 7.4, 0.14 M NaCl, 2.5 mM CaCl_2_,) at a concentration of 1 × 10^6^ cells/ml; 5 μl of Cy5 Annexin-V was added to 1 × 10^5^ cells and incubated for 15 min on ice in the dark. Stained cells were filtered through a 40-μm mesh and analyzed on a FACSCanto II using DIVA Software (Becton Dickinson). Cell debris and duplets were excluded by a combination of light scatter and forward scatter plus width.

### Cell cycle analysis

Cells were incubated with 10 μM BrdU for 2 h at 37 °C and 5% CO_2_. The cells were then fixed in 70% ice-cold ethanol and lysed by incubating first with 2 N HCl and 0.5% Triton X-100 for 30 min and then in 0.1 M Na_2_B_4_O_7_, pH 8.5, for 2 min at room temperature. Nuclei were washed with 0.5% Tween-20, 1% bovine serum albumin (BSA)/PBS and incubated with FITC-labeled anti-BrdU antibody (#347583, Beckton Dickinson) for 30 min at room temperature. Nuclei were stained for DNA content by incubating with 5 μg/ml propidium iodide (PI) for a minimum of 1 h at room temperature. Stained cells were filtered through a 40-μm mesh and analyzed on a FACSCanto II using DIVA Software (Becton Dickinson).

### Chromatin immunoprecipitation

Chromatin immunoprecipitation (ChIP) experiments were performed as previously described with some modifications [51]. In brief, cells were crosslinked either with 1% formaldehyde or in combination with 2 mM EGS (ethylene glycol bis(succinimidyl succinate); ThermoFisher, 21,565). Crosslinked chromatin was sonicated to receive an average fragment size of 500 bp. ChIP was performed with 100 μg of chromatin and 2.5–5 μg HA-tag antibody per IP, and 1% of ChIP material or input material was used for quantitative RT-PCR using specific primers covering Foxf2 binding sites in promoter regions of *Btc* (−450 to −253 from TSS), of *Ereg* (−851 to −654 from TSS), of *Areg* exon2 (+1086 to 1210 from TSS), and of *Noxa* (−696 to −499 from TSS). Primers covering an intergenic region were used as control, and the amplification efficiencies were normalized between the primer pairs. Enrichment of IP/input over IgG background control was calculated and the specificity measured as fold change to an unspecific intergenic region.

### Transcriptome, survival, and metastasis correlation analysis

See Additional file 1.

### Statistical analysis

Statistical analysis and graphs were generated using the GraphPad Prism software (GraphPad Software Inc., San Diego CA). All statistical analyses were performed as indicated by paired or unpaired two-sided *t* test.

## Results

### Foxf2 expression is induced during EMT

We screened for changes in gene expression by DNA oligonucleotide microarray analysis during an EMT in three independent in vitro model systems. First, MTflEcad cells have been derived from a breast tumor of MMTV-Neu transgenic mice [52] in which both E-cadherin alleles were flanked by LoxP recombination sites [53]. Genetic ablation of E-cadherin was achieved by the transient expression of Cre-recombinase (MTΔEcad) [23]. Second, EMT was induced in the human breast cancer cell line MCF7 by downregulation of E-cadherin using stable expression of shRNA [23] and, thirdly, EMT was induced in normal murine mammary epithelial (NMuMG) cells by treatment with TGFβ [54] (Additional file 1: Figure S1A). The forkhead transcription factor Foxf2 was identified as a commonly upregulated gene during EMT in all three experimental systems (Additional file 1: Figure S1B, C). To assess whether Foxf2 is a target of canonical or noncanonical TGFβ signaling, we monitored Foxf2 expression in NMuMG cells stably depleted of Smad4 expression (NMuMG-shSmad4) [48]. Foxf2 mRNA expression levels were significantly reduced in TGFβ-treated NMuMG-shSmad4 cells compared with control cells, indicating that Foxf2 is regulated via canonical Smad4-dependent TGFβ signaling (Additional file 1: Figure S1D).

### Foxf2 is partially required for EMT

**Fig. 1:**
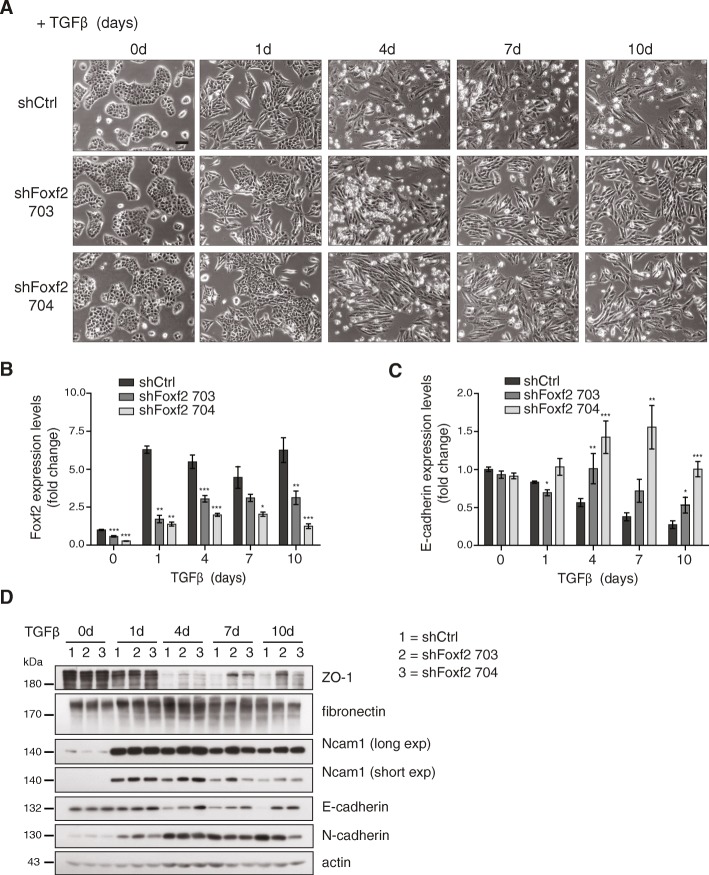
Downregulation of Foxf2 attenuates TGFβ-induced EMT. **a** Phase-contrast micrographs of NMuMG cells stably expressing a control shRNA (shCtrl) or shRNAs against Foxf2 (shFoxf2 703, shFoxf2 704) treated with transforming growth factor (TGF)β for the times indicated. Scale bar = 100μm. **b** Foxf2 knockdown efficiency was determined by quantitative RT-PCR in NMuMG cells stably infected with shCtrl, shFoxf2 703, or shFoxf2 704 and treated with TGFβ for the times indicated. Values were normalized to RPL19 and presented as fold changes compared with untreated shCtrl NMuMG cells. **c** Loss of E-cadherin expression during TGFβ-induced EMT depends on Foxf2. E-cadherin mRNA levels in shFoxf2 and shCtrl-transfected NMuMG cells were determined by quantitative RT-PCR. Values were normalized to RPL19 and reported as fold changes compared with untreated shCtrl NMuMG cells. **d** Knocking down Foxf2 leads to a sustained expression of cell junction components. Immunoblotting analysis for the epithelial markers E-cadherin and ZO-1 as well as the mesenchymal markers Ncam1, N-cadherin, and fibronectin in shFoxf2 knockdown and shCtrl NMuMG cells treated with TGFβ for the times indicated. Actin was used as a loading control. Data are shown as mean ± SEM of three independent experiments. Statistical values were calculated using a paired two-tailed *t* test. *p ≤ 0.05; **p ≤ 0.01; ***p ≤ 0.001

We first assessed whether the expression of Foxf2 is able to induce an EMT by infecting NMuMG cells with lentiviral particles encoding HA-tagged human Foxf2. Although the cells expressed Foxf2 in their nuclei, the cells did not gain an EMT-like phenotype (data not shown). Conversely, to investigate whether the upregulation of Foxf2 expression is required for an EMT, we stably infected NMuMG cells with lentiviral particles expressing two different shRNAs against murine Foxf2 (shFoxf2 #703, shFoxf2 #704). NMuMG-shFoxf2 cells treated with TGFβ apparently changed to a mesenchymal cell morphology, comparable to TGFβ-treated NMuMG-shCtrl cells. However, NMuMG-shFoxf2 cells did not completely lose their tight cell-cell contacts, a key step during an EMT (Fig. 1a). Indeed, quantitative RT-PCR (Fig. 1b, c) and immunoblotting (Fig. 1d) analysis revealed that the shRNA-mediated ablation of Foxf2 expression resulted in a sustained expression of the epithelial adherens and tight junction molecules E-cadherin and ZO-1, whereas the increased expression of the mesenchymal markers fibronectin, Ncam1, and N-cadherin remained unaffected.

To investigate whether the shRNA-mediated depletion of Foxf2 expression affects EMT-associated changes in cell adhesion, cell junctions, and/or cytoskeletal composition, we performed immunofluorescence microscopy analysis for the cell adhesion proteins E-cadherin, N-cadherin, and Ncam1, the tight junction protein ZO-1, the focal adhesion protein paxillin, and actin stress fibers (phalloidin). NMuMG-shFoxf2 cells did not show a classical cadherin switch when treated with TGFβ. In Foxf2-ablated cells, a normal upregulation of the mesenchymal marker N-cadherin was observed, but the expression of the epithelial markers E-cadherin and ZO-1 was partially maintained at the cell membrane, in contrast to shCtrl-expressing cells which showed a bona-fide EMT (Additional file 1: Figure S2A, B). Upregulated expression of Foxf2 during an EMT was also not required for the EMT-associated cytoskeletal reorganization of cortical actin into actin stress fibers, for the upregulation of the mesenchymal marker Ncam1, or for the formation of focal adhesions shown by paxillin staining (Additional file 1: Figure S2B, C). Together, these results indicate that Foxf2 is required for the disruption of cell-cell junctions but not for the induction of a mesenchymal cellular phenotype and mesenchymal marker expression.

### Foxf2 regulates EMT transcriptional regulators and cell migration

**Fig. 2:**
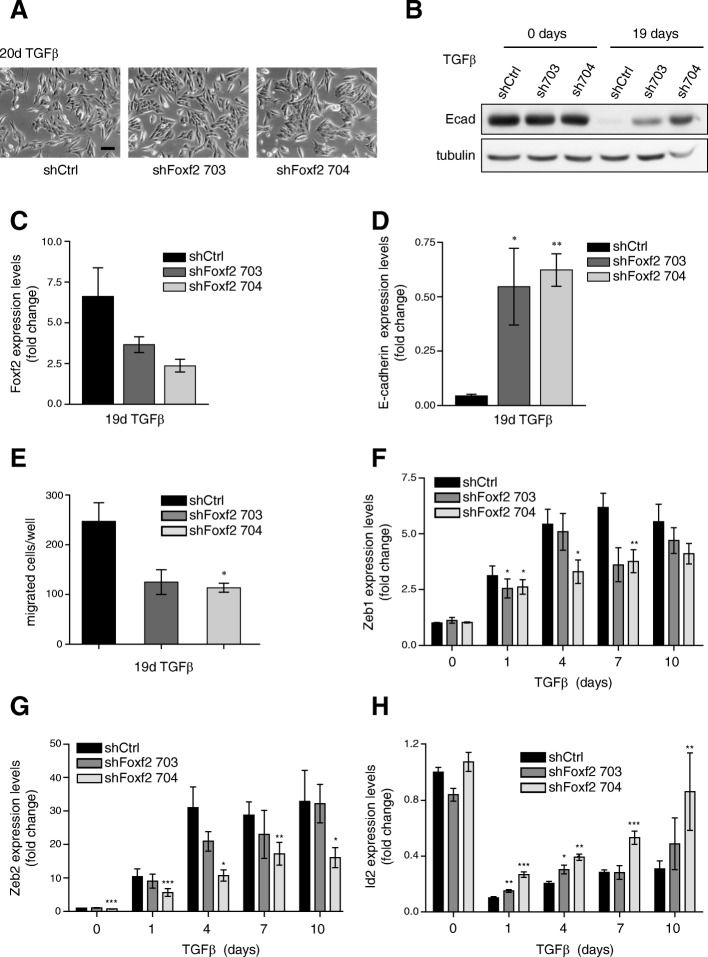
Foxf2 regulates cell migration and the expression of E-cadherin transcriptional repressors. **a** Phase-contrast micrographs of NMuMG cells stably expressing a control shRNA (shCtrl) or a Foxf2-specific shRNA (shFoxf2 703, 704) treated with transforming growth factor (TGF)β for 20 days. Scale bar = 100μm. **b** shRNA-mediated ablation of Foxf2 leads to sustained E-cadherin expression after 19 days of TGFβ treatment shown by immunoblotting analysis in shFoxf2 and shCtrl NMuMG cells. Tubulin was used as a loading control. **c** Foxf2 and **d** E-cadherin mRNA levels were determined by quantitative RT-PCR in shFoxf2 and shCtrl NMuMG cells treated with TGFβ for 19 days. Values were normalized to RPL19 and reported as fold changes compared with untreated shCtrl NMuMG cells. **e** Depletion of Foxf2 leads to reduced cell migration of NMuMG cells treated for 19 days with TGFβ through transwell filters compared with control cells (shCtrl). mRNA levels of **f** Zeb1, **g** Zeb2, and **h** Id2 were determined by quantitative RT-PCR in shFoxf2- and shCtrl-transfected NMuMG cells. Values were normalized to RPL19 and reported as fold changes compared with untreated shCtrl NMuMG cells. Data are shown as mean ± SEM of three independent experiments. Statistical values were calculated using a paired two-tailed *t* test between shCtrl and shFoxf2 cells. *p ≤ 0.05; **p ≤ 0.01; ***p ≤ 0.001

The maintenance of an epithelial morphology and E-cadherin expression in Foxf2-depleted NMuMG cells became even more apparent when treated for 19 to 20 days with TGFβ, a time frame necessary for control NMuMG cells to acquire an EMT stage associated with cellular migration (Fig. 2a–d). Since Foxf2 appeared essential for the loss of E-cadherin expression and the disruption of cell-cell junctions, we assessed whether the loss of Foxf2 expression affected the migratory capabilities of cells. Transwell migration assays revealed a decrease in motility for cells stably expressing shRNAs against murine Foxf2 compared with NMuMG cells expressing control shRNA (Fig. 2e).

The loss of E-cadherin is often attributed to transcriptional dysregulation. Several transcription factors have been identified that are able to repress E-cadherin gene expression, among them the zinc-finger-homeodomain transcription factors Zeb1 and Zeb2 [55–57]. Quantitative RT-PCR analysis revealed that shRNA-mediated ablation of Foxf2 expression during a TGFβ-induced EMT in NMuMG cells attenuated the upregulation of Zeb1 and Zeb2 expression observed in shCtrl-transfected cells (Fig. 2f, g). Inhibitors of differentiation (Ids) act as positive regulators of proliferation and as negative regulators of differentiation. The Id proteins lack a DNA-binding motif and inhibit, for example, E2A-dependent suppression of the E-cadherin promoter [58]. Consistent with the sustained expression of E-cadherin, ablation of Foxf2 in TGFβ-treated NMuMG cells interfered with the downregulation of Id2 expression during an EMT (Fig. 2h).

The loss of Foxf2 maintained E-cadherin expression during a TGFβ-induced EMT in NMuMG cells in a similar manner as the loss of the major transcriptional repressor of E-cadherin expression, Zeb1 (Additional file 1: Figure S3A, B). Also comparable to the siRNA-mediated ablation of Zeb1 expression, loss of Foxf2 expression resulted in the upregulated expression of the miR-200 family members miR-200a-3p, miR-200b-3p, and miR-429-3p (Additional file 1: Figure S3C). On the other hand, both Foxf2 and Zeb1 are predicted targets of miR-200 family members, and ectopic expression of the miR-200 family members miR-200b-3p, miR-200c-3p, and miR-429-3p downregulated both transcription factors (Additional file 1: Figure S3D, E). Consistent with a regulatory role of Foxf2 on the expression of Zeb family proteins, Foxf2 upregulation after 1 day of TGFβ treatment of NMuMG cells was followed by the induction of Zeb1 and Zeb2 expression, leading to a continuous downregulation of E-cadherin (Additional file 1: Figure S3F). These results indicate tight control of E-cadherin expression by a double-negative feedback loop between Foxf2 and miR-200 family members, as well as regulation of the expression of known targets of miR200 family members and transcriptional repressors of E-cadherin expression, such as Zeb1, Zeb2, and Id2. Similarly, Foxf2 is essential for the proper downregulation of E-cadherin and the regulation of Zeb2 and Id2 during a TGFβ-induced EMT of Py2T murine breast cancer cells that have been derived from a tumor of the MMTV-PyMT mouse model of breast cancer (Additional file 1: Figure S3G).

In conclusion, the upregulation of Foxf2 in NMuMG cells undergoing an EMT is essential for the transcriptional repression of E-cadherin, for the disruption of cell-cell adhesions, and for EMT-associated cell migration, yet has only minor effects on the induction of mesenchymal marker expression.

### Foxf2 regulates cell death and survival pathways

**Fig. 3:**
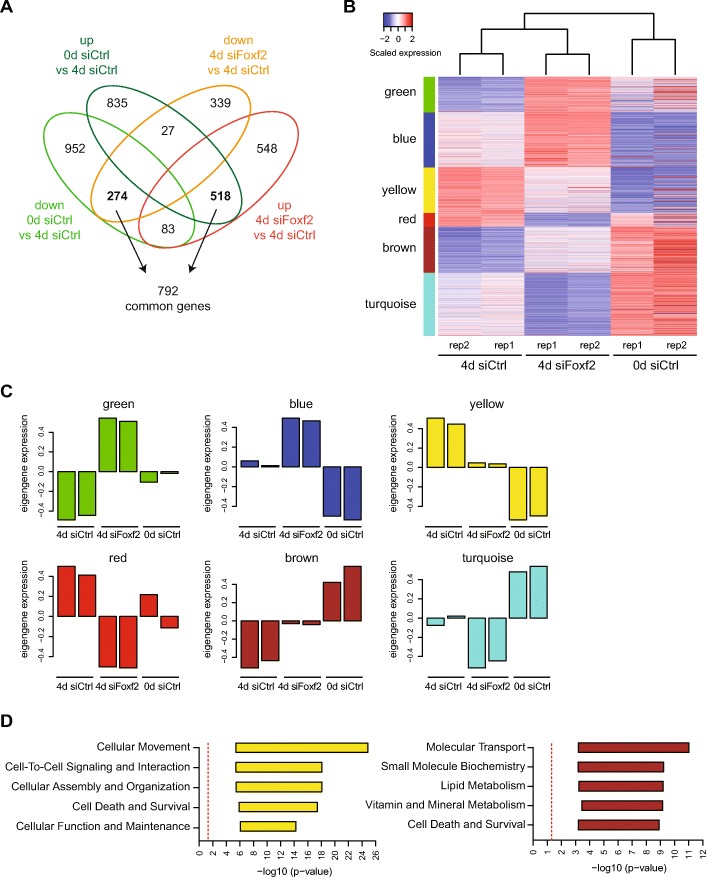
RNA sequencing analysis of Foxf2-dependent gene expression. **a** Venn diagram illustrating the overlap of significantly differentially expressed genes (*p* adjusted < 0.05, abs(log2 fold change) ≥ 1) between the epithelial and mesenchymal state (0 days vs 4 days siRNA control (siCtrl)) or for the Foxf2 perturbation (4 days siFoxf2 vs 4 days siCtrl). Highlighted in bold are genes whose change during EMT is reversed by Foxf2 knockdown. **b** Heatmap of gene expression from RNA-sequencing of epithelial control samples (untreated siCtrl NMuMG cells) and of siCtrl- or siFoxf2-treated NMuMG cells in the presence of TGFβ for 4 days. The sample order in the heatmap was obtained from an unsupervised hierarchical clustering, while rows (genes) were arranged according to the gene signatures derived from WGCNA. **c** The eigengene expression of the different gene signatures derived from WGCNA (see **a**) illustrates a general expression trend of all genes belonging to a gene signature. **d** IPA analysis of the brown and yellow gene signature. Shown in the bar plot are the significance ranges of pathways belonging to the most significantly enriched categories. The dotted red line indicates a *p* value of 0.05. Differential gene expression and gene signature memberships are reported in Additional file 1 (Table S1 and S2, respectively)

To identify the actual genes and signaling pathways that are regulated by Foxf2 during an EMT, we performed gene expression profiling by RNA sequencing of NMuMG cells that were transfected with control siRNA (siCtrl; epithelial state) or with siCtrl or siRNA targeting Foxf2 (siFoxf2) in the presence of TGFβ for 4 days (mesenchymal state). We found 1789 genes to be differentially expressed at least twofold upon Foxf2 knockdown compared with siRNA control at 4 days of TGFβ treatment, and 2689 genes were significantly changed upon induction of EMT comparing siCtrl in the absence or presence of TGFβ (siCtrl 0 days vs 4 days TGFβ; ΕΜΤ). In total, 792 genes were commonly regulated by the loss of Foxf2 expression and by the induction of an EMT with TGFβ (Fig. 3a; Additional file 1: Table S1). Unsupervised hierarchical clustering revealed that Foxf2-deficient cells treated with TGFβ more closely resembled the mesenchymal state of TGFβ-treated control cells, however they formed a separate clustering arm (Fig. 3b). To study in more detail which transcripts were specifically altered compared with the mesenchymal and epithelial control states, gene expression signatures were generated using weighted gene coexpression network analysis (WGCNA). Six different gene expression signatures were extracted, with the yellow and the brown signatures summarizing genes from an intermediate mesenchymal state (Fig. 3b; Additional file 1: Table S2). The EMT-induced expression of the genes in the yellow signature was strongly reduced by the ablation of Foxf2 knockdown. Conversely, the EMT-repressed expression of the genes in the brown signature was blocked by the loss of Foxf2 (Fig. 3c).

**Fig. 4:**
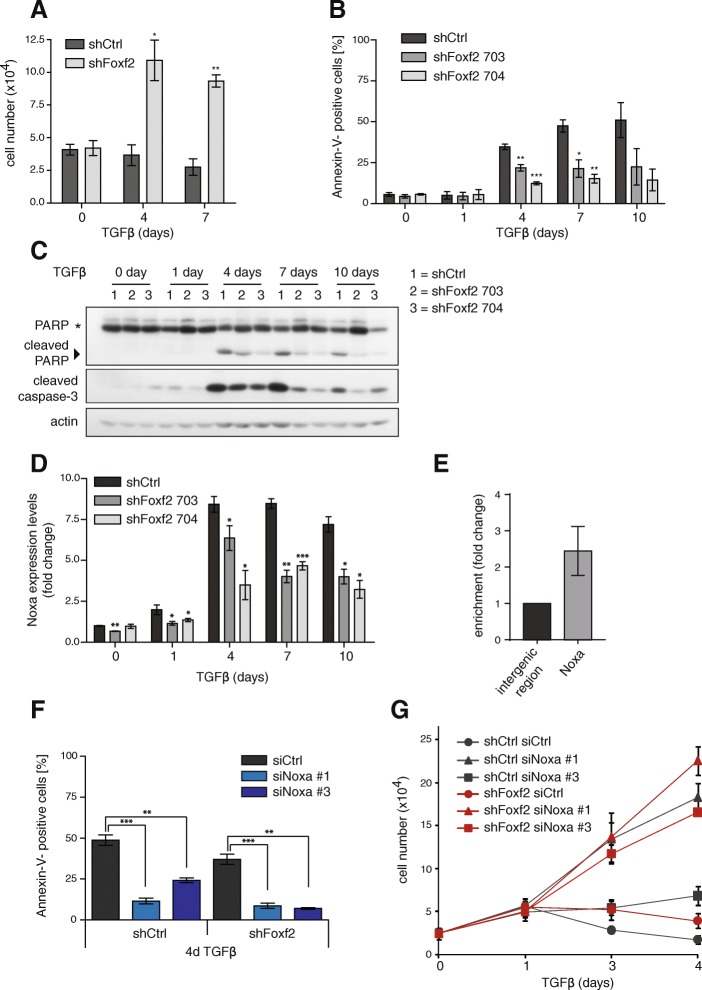
Depletion of Foxf2 attenuates TGFβ-induced apoptosis and the expression of the proapoptotic protein Noxa. **a** Downregulation of Foxf2 promotes cell proliferation. shFoxf2- and shCtrl-expressing NMuMG cells were treated with transforming growth factor (TGF)β for the times indicated and counted using a Neubauer chamber. **b** Foxf2 depletion decreases apoptosis during TGFβ-induced EMT. shFoxf2- and shCtrl-expressing NMuMG cells were treated with TGFβ for the times indicated, and apoptosis was detected by Annexin-V staining and flow cytometry analysis. **c** TGFβ induces classical caspase-mediated apoptosis dependent on the upregulation of Foxf2. Immunoblotting analyses of the same experiment as shown in Fig. 1d for cleaved caspase-3 and PARP in shFoxf2- and shCtrl-expressing NMuMG cells treated with TGFβ for the times indicated. Actin was used as a loading control. **d** Knockdown of Foxf2 attenuates the upregulation of Noxa expression. Noxa mRNA levels in shFoxf2- and shCtrl-expressing NMuMG cells treated with TGFβ for the times indicated were determined by quantitative RT-PCR. Values were normalized to RPL19 and presented as fold changes compared with untreated shCtrl NMuMG cells. **e** Foxf2 regulates Noxa expression by direct transcriptional activation. Chromatin immunoprecipitation of HA-tagged Foxf2 was performed either on Foxf2-expressing or control NMuMG cells treated for 2 days with TGFβ. Immunoprecipitated DNA fragments were quantified by quantitative PCR using primers covering base pairs −696 to −499 of the *noxa* promoter region. Enrichment (IP/input) for specific primers was calculated relative to primers covering an intergenic region. **f** Noxa depletion significantly decreases TGFβ-induced apoptosis. shFoxf2- and shCtrl-expressing NMuMG cells were transfected with control siRNA (siCtrl) and two different siRNAs specific for murine Noxa (siNoxa #1, siNoxa #3) and incubated with TGFβ for 4 days. The extent of apoptosis was measured by Annexin-V staining and flow cytometry. **g** The impairment of Noxa expression leads to increased cell proliferation. shFoxf2- and shCtrl-expressing NMuMG cells were transfected with control siRNA (siCtrl) and two different siRNAs specific for murine Noxa (siNoxa #1, siNoxa #3) and incubated with TGFβ for the times indicated. Cell numbers were determined using a Neubauer chamber. Results show the mean ± SEM of three independent experiments. Statistical values were calculated using paired/unpaired two-tailed *t* test. *p ≤ 0.05; **p ≤ 0.01; ***p ≤ 0.001

Pathway enrichment analysis using ingenuity pathway analysis (IPA) revealed major functions of the yellow signature-associated genes in cellular movement and cell-cell signaling and interaction, functions that can be attributed to the loss of E-cadherin expression as described above (Fig. 3d). On the other hand, the brown signature was found to be associated with molecular transport and metabolism (lipid, vitamin, and mineral metabolism) pathways (Fig. 3d). Interestingly, both up- and downregulated EMT signatures that are also affected by Foxf2 knockdown (brown and yellow signatures) were enriched in pathways describing cell death and survival, indicating a regulatory role of Foxf2 in these processes (Fig. 3d). Indeed, NMuMG cells stably expressing shRNA against Foxf2 showed significantly less TGFβ-mediated growth inhibition compared with shCtrl-transfected cells, and the cell number increased significantly compared with shCtrl-expressing cells (Fig. 4a).

### Foxf2 affects apoptosis by regulating the expression of Noxa

To assess whether the increase in cell numbers was due to increased proliferation or decreased cell death, we compared the rates of apoptosis and proliferation by Annexin-V staining and BrdU incorporation and PI staining, respectively, in NMuMG cells treated with TGFβ and depleted or not for Foxf2 expression. The loss of Foxf2 significantly reduced the levels of apoptosis when compared with control cells (Fig. 4b), while only a moderate difference in the number of cycling cells was observed (Additional file 1: Figure S4A). The lack of Foxf2 reduced caspase-dependent programmed cell death, as the levels of cleaved caspase-3 and its downstream cleavage target poly-(ADP-ribose) polymerase (PARP) were diminished upon knockdown of Foxf2 (Fig. 4c). In summary, the results show that the increased expression of Foxf2 during EMT is critical for promoting TGFβ-induced cell death.

TGFβ has been shown to act as a tumor suppressor in the early stages of tumorigenesis by inducing the expression of cell cycle inhibitors and proapoptotic factors [59]. Differential gene expression analysis between TGFβ-treated control and Foxf2-deficient NMuMG cells revealed a substantial regulation of the BH3-only factor Noxa by Foxf2, which was confirmed by quantitative RT-PCR analysis (Fig. 4d). ChIP experiments with NMuMG cells expressing HA-tagged Foxf2 treated for 2 days with TGFβ demonstrated a weak direct binding of Foxf2 to the *noxa* gene promoter, suggesting additional indirect regulatory mechanisms (Fig. 4e). As the upregulation of Foxf2 is necessary for TGFβ-induced Noxa expression, we next assessed whether loss of Noxa is sufficient to prevent apoptosis in TGFβ-treated NMuMG cells. Noxa expression in NMuMG cells was ablated by transient transfection of two different siRNAs (siNoxa #1, siNoxa #3) or control siRNA (siCtrl) in shFoxf2- and shCtrl-expressing cells. Following the reduction of Noxa mRNA levels in siNoxa #1- and #3-treated cells (Additional file 1: Figure S4B), apoptosis was attenuated and cell growth inhibition was compensated in TGFβ-treated cells (Fig. 4f, g; Additional file 1: Figure S4C). These results demonstrate that depletion of Noxa is sufficient to protect NMuMG cells from TGFβ-induced apoptosis. Together, these data indicate that Foxf2 mediates TGFβ-induced apoptosis by the transcriptional activation of the proapoptotic protein Noxa.

### Foxf2 promotes TGFβ-induced growth arrest by repressing EGF receptor signaling

**Fig. 5:**
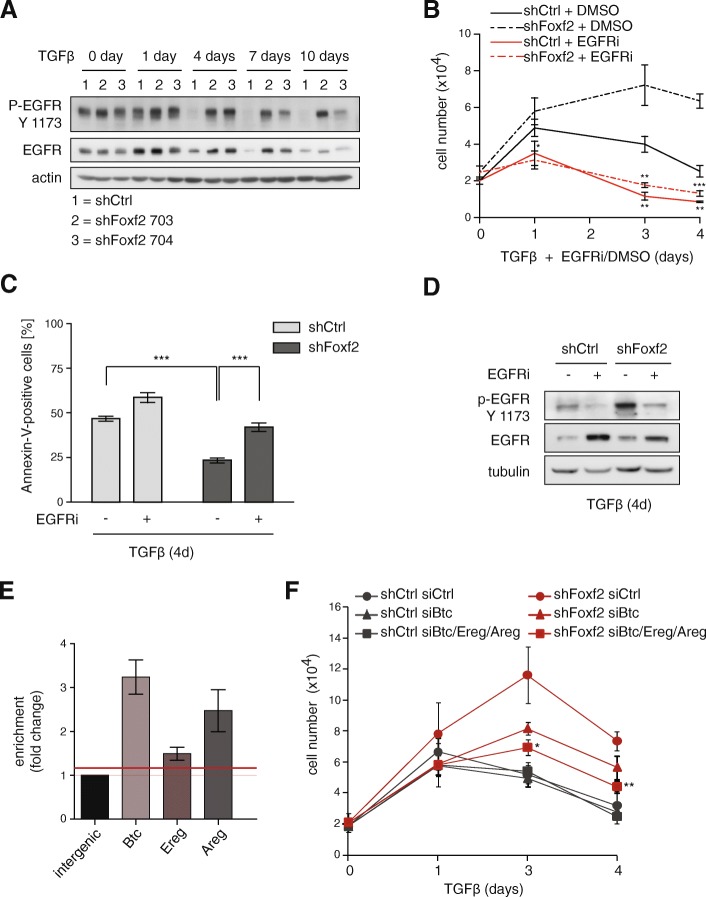
Inhibition of EGFR signaling increases apoptosis in Foxf2-depleted cells. **a** Depletion of Foxf2 leads to sustained epidermal growth factor receptor (EGFR) activation. Immunoblotting analysis of the phosphorylation status of EGFR and total EGFR protein levels in shFoxf2- and shCtrl-expressing NMuMG cells treated with transforming growth factor (TGF)β for the times indicated. Actin was used as a loading control. **b**–**d** NMuMG cells expressing shRNA specific for Foxf2 or control shRNA were treated with TGFβ and AG1478 (EGFR inhibitor (EGFRi)) or control solvent (dimethyl sulfoxide (DMSO)) for the indicated times. **b** Cell numbers were determined using a Neubauer chamber. **c** EGFR inhibition significantly increases apoptosis in Foxf2 knockdown cells. Apoptosis was detected by Annexin-V staining and flow cytometry. **d** Treatment with AG1478 decreases EGFR activation in shFoxf2 cells to a similar extent as seen in TGFβ-treated NMuMG cells expressing control shRNA. Immunoblotting analysis for EGFR phosphorylation and total EGFR levels is shown. Tubulin was used as a loading control. **e** Foxf2 regulates the expression of EGFR ligands by direct transcriptional repression. Chromatin immunoprecipitation of Foxf2 was performed either on HA-Foxf2 expressing or control NMuMG cells treated for 2 days with TGFβ. Immunoprecipitated DNA fragments were quantified by quantitative PCR using primers covering base pairs −450 to −253 of the *Btc* promoter region, base pairs −851 to −654 of the *Ereg* promoter, and base pairs +1086 to 1210 of the *Areg* exon 2. Enrichment (IP/input) for specific primers was calculated relative to primers covering an intergenic region. **f** Individual depletion of Btc or combined depletion of betacellulin (Btc), epiregulin (Ereg), and amphiregulin (Areg) reduces cell numbers in shFoxf2- but not in shCtrl-expressing NMuMG cells in the presence of TGFβ for 4 days. Cell numbers were determined using a Neubauer chamber. Data are shown as mean ± SEM of at least three independent experiments. Statistical values were calculated using a paired/unpaired two-tailed *t* test. *p ≤ 0.05; **p ≤ 0.01; ***p ≤ 0.001

As well as activating transcription of the *noxa* gene and inducing apoptosis, we investigated whether Foxf2 regulates any prosurvival signaling pathway. EGF receptor (EGFR) family members are known to provide protection from TGFβ-induced cell cycle arrest and apoptosis by activating the PI3K pathway [60, 61]. Indeed, immunoblotting analyses revealed that the levels of activated (tyrosine 1173-phosphorylated) forms of EGFR were higher in Foxf2-depleted NMuMG cells compared with control NMuMG cells when treated with TGFβ (Fig. 5a).

Previously, we have reported that survival of NMuMG cells undergoing a TGFβ-induced EMT depends on activated EGFR signaling [20]. We thus investigated whether inhibition of EGFR signaling impaired the antiapoptotic effect of Foxf2 depletion. Towards this aim, shFoxf2- and shCtrl-transfected NMuMG cells were treated with the EGFR inhibitor (EGFRi) AG1478 during TGFβ treatment, and cell growth and rates of apoptosis were determined. Combined treatment with EGFRi and TGFβ for 4 days led to a significantly reduced growth in shFoxf2-expressing NMuMG cells compared with the solvent (dimethyl sulfoxide (DMSO))-treated shFoxf2-expressing NMuMG cells (Fig. 5b; Additional file 1: Figure S5A). In addition, treatment of shFoxf2-expressing NMuMG cells with AG1478 increased apoptosis to a similar extent as observed in shCtrl-expressing cells (Fig. 5c). The extent of apoptosis thereby correlated with the levels of EGFR inhibition (Fig. 5d). This result indicates that TGFβ-resistant growth of Foxf2 knockdown cells relies on the activation of EGFR survival signaling.

To investigate how Foxf2 influences EGFR activation, we assessed whether the expression of EGFR ligands was affected by the modulation of Foxf2 expression. Gene expression profiling and validation by quantitative RT-PCR revealed that the EGFR-ligands betacellulin (Btc), amphiregulin (Areg), and (moderately) epiregulin (Ereg) showed sustained expression upon knockdown of Foxf2 during an EMT in both NMuMG and Py2T cells (Additional file 1: Figure S5B, C). Promoter binding prediction programs indicated a potential direct binding of Foxf2 to the *Btc* promoter (data not shown). ChIP followed by quantitative PCR of HA-tagged Foxf2 in NMuMG cells during TGFβ-induced EMT revealed a direct binding of Foxf2 to the *Btc* promoter region, to a regulatory region in exon 2 of the *Areg* gene, and with less efficiency to the *Ereg* promoter region (Fig. 5e).

To assess whether Btc, Areg, or Ereg were responsible for the stimulation of EGFR and increased cell survival of Foxf2-depleted cells, NMuMG cells stably expressing shRNA against Foxf2 or a control shRNA were transiently transfected with siRNAs against Btc or with a mix of siRNAs against Btc, Areg, and Ereg and treated with TGFβ. The efficiency of Btc or combined Btc/Areg/Ereg ablation was determined by quantitative RT-PCR (Additional file 1: Figure S5D, E). Knockdown of Btc alone or in combination with the other two family members resulted in reduced cell growth in shFoxf2-expressing cells when treated with TGFβ (Fig. 5f). These results indicate that attenuation of EGFR activation by siRNA-mediated depletion of its ligands abrogates the survival benefit of Foxf2 depletion. We conclude that TGFβ-induced Foxf2 expression represses the transcriptional activation of the *Btc* and *Areg* genes, resulting into a reduced expression of these EGFR ligands and a repression of EGFR survival signaling.

### Foxf2 expression correlates with poor prognosis in patients

**Fig. 6:**
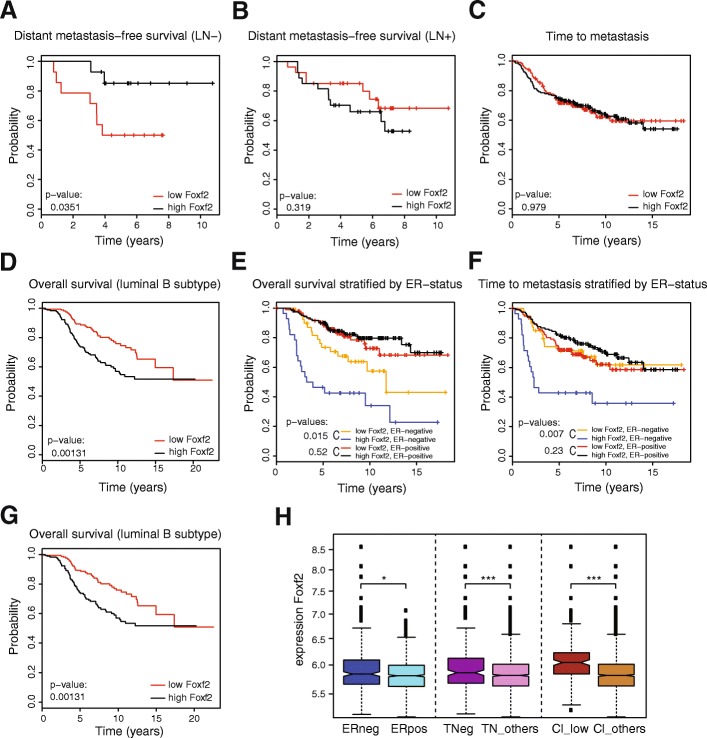
High Foxf2 expression correlates with good prognosis in early-onset breast cancer patients but with poor prognosis in late-stage estrogen receptor-negative and luminal B breast cancer patients. **a**,**b** Statistical analysis of the Memorial Sloan-Kettering Cancer Center (Minn) database. Tumors were divided into high and low Foxf2-expressing groups based on the median expression of Foxf2 mRNA. Expression of Foxf2 was correlated with distant metastasis free survival in lymph node-negative (LN^–^) tumors (*n* = 28) or LN^+^ tumors (*n* = 54). Foxf2 expression is predictive for metastasis incidence in LN^–^ tumors (**a**), where low Foxf2 expression correlates with early metastases onset (*p* value = 0.0351), but not for LN^+^ tumors (**b**). **c**–**f** Statistical analysis of the Netherlands Cancer Institute (NKI295) database. Tumors were divided into high and low Foxf2-expressing groups based on the relative expression of Foxf2 compared with the tumor pool (log fold change = 0). Expression of Foxf2 was correlated with time to metastasis (**c**, **f**) or overall survival (**d**, **e**) either in all tumors analyzed (**c**; *n* = 288) or stratified for estrogen receptor (ER) expression (**e**, **f**; ER-negative: *n* = 68; ER-positive: *n* = 220). Foxf2 expression does not correlate with tumor metastasis in the total tumor pool (**c**), but high Foxf2 expression is predictive for overall patient survival in luminal subtype B (**d**; *p* = 0.00131) and ER-negative tumors (**e**; *p* = 0.0154), respectively. High Foxf2 expression levels also correlate with an early onset of metastasis in ER-negative patients (**f**; *p* = 0.007). **g** ,**h** Statistical analysis of the Metabric database (*n* = 1298 tumors). **g** Tumors were divided into high and low Foxf2-expressing groups based on the median expression of Foxf2 mRNA. In the luminal subtype B tumors (*n* = 352), Foxf2 expression is predictive for overall survival (*p* value of the likelihood-ratio test). **h** Expression of Foxf2 across multiple different tumor subtypes shows significantly increased levels in ER-negative (ERneg) vs ER-positive (ERpos) tumors, in triple negative (TNeg), and in claudin-low (Cl_low) compared with all other tumors (*p* value of the Kruskal-Wallis test; *p ≤ 0.05; ***p ≤ 0.001)

Cancer-associated gene expression profiling has emerged as an appropriate tool to predict the relapse risk and to identify genes that mediate disease progression. To investigate whether Foxf2 expression is predictive for tumor progression or metastasis formation, we analyzed a breast cancer database of the Memorial Sloan-Kettering Cancer Center (MSKCC), published by Minn et al. [62]. This “Minn” database consists of microarray gene expression analysis of tumor samples from 82 patients with advanced breast cancer (T2–T4). The tumors were divided into two groups according to the log expression levels relative to the median expression of the investigated gene. The low and high Foxf2-expressing groups were further stratified for lymph node (LN) metastasis status. Interestingly, low Foxf2 expression significantly correlated with early distant metastasis formation in lymph node-negative (LN^–^) tumors, whereas the opposite tendency was found in tumors of patients that were positive for lymph node metastasis (LN^+^) (Fig. 6a, b).

To further substantiate a potential correlation between Foxf2 expression and patient survival, we analyzed the Netherlands Cancer Institute (NKI295) breast cancer database for Foxf2 expression [63]. The NKI295 database consists of microarray gene expression analysis of tumor samples from 295 patients with early-stage breast cancer (stage I or stage II primary breast carcinomas). Although Foxf2 expression was not predictive for metastasis formation or survival in the total patient pool (Fig. 6c), high expression of Foxf2 correlated with poor overall survival in patients with luminal subtype B breast cancer (Fig. 6d). High expression of Foxf2 in tumors with negative estrogen receptor (ER) status correlated with high significance of early metastasis onset as well as poor overall survival (Fig. 6e, f). Similarly, in a large tumor collection from the Metabric consortium [64, 65], high Foxf2 expression predicted worse survival in the luminal B breast cancer subtype (Fig. 6g). Interestingly, Foxf2 expression was significantly higher in more aggressive tumor subtypes, such as ER^–^ compared with ER^+^, triple-negative compared with all other subtypes, and in claudin-low tumors (the breast cancer subtype associated with an EMT signature), compared with all others (Fig. 6h).

Together, the expression of Foxf2 in human patient samples and its prediction for clinical outcome reflect the dual function of Foxf2 observed in our in-vitro studies. Foxf2 may function as a tumor suppressor in early cancer development by promoting apoptosis, hence showing a poor prognosis in LN^–^ patients with low Foxf2 expression. More advanced tumors with high Foxf2 expression correlate with shorter metastasis-free survival, supporting a role of Foxf2 in cancer cell invasion and metastasis formation.

## Discussion

Overcoming the growth inhibitory effect of TGFβ during the early stages of tumorigenesis as well as the conversion of TGFβ-mediated growth inhibition into TGFβ-induced tumor progression are fundamental processes during primary tumor growth and metastasis formation [1, 66]. Thus, understanding the mechanisms underlying this dual role of TGFβ in cancer progression and the strategies of cancer cells to circumvent TGFβ-induced apoptosis may offer new opportunities for novel cancer therapies.

**Fig. 7:**
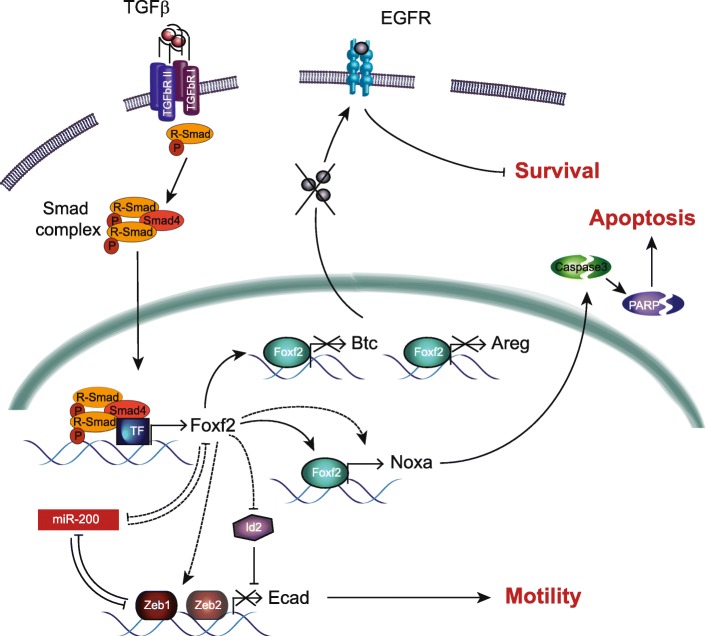
Schematic model of the dual role of Foxf2 during TGFβ-induced EMT. Receptor activation by transforming growth factor (TGF)β leads to phosphorylation of receptor-bound Smads with subsequent Smad complex formation. The trimeric complex enters the nucleus to activate the expression of TGFβ target genes, such as the forkhead transcription factor Foxf2. Subsequently, Foxf2 directly represses the transcription of the epidermal growth factor (EGFR) ligands betacellulin (Btc) and amphiregulin (Areg), which leads to reduced EGFR-mediated survival signaling. Furthermore, Foxf2 transcriptionally activates the proapoptotic gene *Noxa*, resulting in the induction of caspase-dependent apoptosis. On the other hand, downregulation of E-cadherin (Ecad) is modulated by Foxf2-dependent upregulation of Zeb1/Zeb2, downregulation of Id2, and a double-negative feedback loop between Foxf2 and the miR-200 family, leading to the disruption of adherens junctions and the subsequent increase in cell migration

Here, we have employed nontransformed NMuMG cells and Py2T murine breast cancer cells to delineate the molecular mechanisms underlying a TGFβ-induced EMT, including overcoming TGFβ-induced resistance to apoptosis and the acquisition of invasive properties. We report a dual function of the transcription factor Foxf2, acting as a tumor suppressor by promoting apoptosis and by repressing survival, while exerting protumorigenic activity at later stages of tumor progression, such as a promigratory function by inducing the disruption of cell-cell adhesion. Foxf2 is upregulated via the canonical TGFβ pathway, and gain of function studies in NMuMG cells reveal that its expression is not sufficient to induce an EMT. However, loss of function studies demonstrate that Foxf2 is essential for the disruption of cell junctions and the repression of epithelial marker expression but not for the gain of mesenchymal marker expression. Notably, Foxf2 is crucial for TGFβ-mediated cell death by the transcriptional activation of the *Nox*a gene, encoding for a BH3-only proapoptotic factor, and the subsequent induction of caspase-dependent apoptosis. Moreover, Foxf2 directly represses transcription of the *Btc* and *Areg* genes, encoding for ligands of EGFR, and thus attenuates EGFR-mediated survival signaling (Fig. 7).

The failure of Foxf2-depleted cells to disrupt tight and adherens junctions exemplifies its indispensable role in the acquisition of an invasive cell morphology. Loss of E-cadherin is an early event during EMT resulting in the disruption of the polarity complex, a prerequisite for the dissociation and invasion of cancer cells [67–69]. Direct transcriptional repression has emerged as one common regulatory mechanism of E-cadherin expression in various cancer types [70, 71]. Here we demonstrate that, by mediating the TGFβ-induced upregulation of the transcriptional repressors Zeb1 and Zeb2 as well as the repression of Id2, Foxf2 mediates the transcriptional downregulation of E-cadherin and consequently the disruption of cell-cell adhesion. In addition, we show that Foxf2 also affects the expression of the miR-200 family which are potent EMT-repressing noncoding RNAs that target Zeb1 and Zeb2 transcripts [72–74]. Interestingly, the expression of Foxf2 and miR-200 are controlled in a double-negative feedback loop, similar to the well-studied Zeb1-miR200 loop. The mechanism by which Foxf2 regulates the expression of these transcription (co)factors remains elusive, but the presence of putative Foxf2 binding sites in the promoter region of Zeb1 and Zeb2 (data not shown) and data from Kundu et al. [75] in nonsmall-cell lung cancer (NSCLC) cells suggest a direct regulatory mechanism, thus ensuring the downregulation of cell-cell junctions at multiple levels (Fig. 7). Moreover, consistent with our finding that Foxf2 predicts poor survival in a subset of breast cancer patients, our results identify Foxf2 as a promigratory and prometastatic factor (Fig. 7).

Our results also show that Foxf2 is essential for TGFβ-mediated apoptosis. The reduction of TGFβ-induced apoptosis in Foxf2-deficient cells is a consequence of the loss of the transcriptional activation of *Noxa* gene expression. Noxa has been shown to be critical in fine-tuning cell death decisions via degradation of the prosurvival molecule Mcl1 [76]. Noxa has been identified as a primary p53 target gene; however, oncogenic stress, such as irradiation and hypoxia, results in efficient induction of Noxa also in the absence of p53 [77, 78]. Our findings identify Foxf2 as a novel transcriptional activator of the tumor suppressor Noxa (Fig. 7).

Besides triggering apoptosis via regulation of Noxa expression, Foxf2 is also involved in the negative regulation of survival signals by the transcriptional repression of the EGFR ligands betacellulin (Btc), amphiregulin (Areg), and, to a lesser degree, epiregulin (Ereg). Although the regulatory effect of Foxf2 on the transcription level of the individual EGFR ligands is moderate (Additional file 1: Figure S5B, C), a cumulative effect by the simultaneous modulation of Btc, Areg, and Ereg expression enables a significant shift towards less EGFR signaling (Fig. 5a). Reduced EGFR signaling leads to a reduction in EGFR phosphorylation and, hence, reduced PKB activation. Blocking EGFR signaling has been shown to amplify the apoptotic response to TGFβ [61]. Here, we show that both pharmacological receptor inhibition as well as the combined reduction of the expression of the EGFR ligands Btc, Areg, and Ereg increased TGFβ-induced apoptosis in Foxf2-depleted NMuMG cells. These results indicate that, in addition to Noxa regulation, Foxf2 mediates its apoptotic effect by blocking EGFR-mediated survival signaling (Fig. 7).

To support the importance of a Foxf2 function during tumor development and progression, we have performed correlation studies for Foxf2 on three different breast cancer databases [62–64]. Analysis of a lymph node-negative stratified patient subset demonstrates that low Foxf2 expression significantly correlates with early metastasis formation. Comparably, low Foxf2 expression has been recently reported to correlate with early-onset metastasis and poor prognosis in breast cancer patients [43]. Interestingly, the opposite is found in more progressive breast cancers, where high Foxf2 expression correlates with poor prognosis. Stratification for luminal subtype B or for ER status reveals a highly significant correlation of high Foxf2 expression and early metastasis onset, concomitant with reduced overall survival. ER^–^ tumors represent highly aggressive breast cancer subtypes. In addition, Foxf2 transcript levels are increased in more aggressive ER^–^, in triple negative, and in the EMT-like, claudin-low breast cancer subtypes. Together, our findings identify high levels of Foxf2 as a marker for good prognosis in early noninvasive stages of tumor development, but with poor prognosis in malignant stages [79]. These findings substantiate the dual role of Foxf2 in cancer patients.

## Conclusions

In our study, we demonstrate a dual role for the transcription factor Foxf2. It induces proapoptotic and represses antiapoptotic genes and, thus, acts as a tumor suppressor, likely with the help of specific cofactors. On the other hand, it induces the expression of EMT-inducing genes and thus exerts prometastatic functions to cells that have overcome the apoptotic crisis and undergone EMT. The role of Foxf2 in pre- and post-EMT cells reflects the well-studied dual role of TGFβ in cancer progression. Our results also substantiate findings in knockout mouse models where Foxf2 was found to play an important role in EMT-associated developmental processes and maintenance of the epithelial-mesenchymal structure in lung and gut tissues [38, 39]. Hence, fine-tuning of the expression of Foxf2 and its cofactors could be pivotal during carcinogenesis, and insights into its regulation and molecular function are critical for the design of novel therapeutic strategies.

## Additional file

Additional file 1: **Figure S1.** Foxf2 expression is upregulated during EMT via the canonical Smad pathway. The increased expression of Foxf2 during an EMT was assessed in normal murine mammary gland epithelial cells (NMuMG) and in murine and human breast cancer cells. **Figure S2.** Foxf2 is required for TGFβ-induced disruption of adherens junctions. RNAi-mediated ablation prevents an EMT as visualized by immunofluorescence staining for epithelial and mesenchymal markers. **Figure S3.** Foxf2 regulates the expression of Zeb1, Zeb2, Id2, and members of the miR-200 family as determined by quantitative RT-PCR of their expression during an EMT in the absence of presence of Foxf2. **Figure S4.** Foxf2 regulates Noxa expression and thus affects cell proliferation and apoptosis. Foxf2 regulated the expression of Noxa, and siRNA-mediated depletion of Noxa prevented an increase in cell death induced by the loss of Foxf2 expression as assessed by quantitative RT-PCR. **Figure S5.** EGF ligand-mediated EGF receptor signaling overcomes Foxf2-controlled cell survival. Foxf represses the expression of EGF receptor ligands as assessed by quantitative RT-PCR. **Supplementary material and methods.** Detailed information is given on the antibodies and reagents, on biochemical and cell biological methods, and on RNA sequencing and bioinformatics analysis used in the study. **Table S1.** Excel file summarizing the differential expression analysis (siFoxf2 to siCtrl after 4 days TGFβ treatment or siCtrl with vs without TGFβ for 4 days) of all transcripts detected with RNA-sequencing. **Table S2.** Excel file showing the list of genes belonging to the different gene signatures (modules) and the strength of their modular membership (kME values). (ZIP 14675 kb)

## Abbreviations

Areg: Amphiregulin
Btc: Betacellulin
ChIP: Chromatin immunoprecipitation
EGFR: Epidermal growth factor receptor
EGFRi: EGFR inhibitor
EMT: Epithelial to mesenchymal transition
Ereg: Epiregulin
Foxf2: Forkhead box protein F2
Ids: Inhibitors of differentiation
IPA: Ingenuity pathway analysis
MET: Mesenchymal to epithelial transition
NMuMG: Normal murine mammary gland
PARP: Poly-(ADP-ribose) polymerase
TGF: Transforming growth factor
WGCNA: Weighted gene coexpression network analysis
