# Supplementary material for: Foxf2 plays a dual role during transforming growth factor beta-induced epithelial to mesenchymal transition by promoting apoptosis yet enabling cell junction dissolution and migration
Source: Breast Cancer Res. 2018 Oct 1;20:118. doi: 10.1186/s13058-018-1043-6 (PMC6167826; doi:10.1186/s13058-018-1043-6)
Supplement: Supplementary file 1 — Figure S1. Foxf2 expression is upregulated during EMT via the canonical Smad pathway. The increased expression of Foxf2 during an EMT was assessed in normal murine mammary gland epithelial cells (NMuMG) and in murine and human breast cancer cells. Figure S2. Foxf2 is required for TGFβ-induced disruption of adherens junctions. RNAi-mediated ablation prevents an EMT as visualized by immunofluorescence staining for epithelial and mesenchymal markers. Figure S3. Foxf2 regulates the expression of Zeb1, Zeb2, Id2, and members of the miR-200 family as determined by quantitative RT-PCR of their expression during an EMT in the absence of presence of Foxf2. Figure S4. Foxf2 regulates Noxa expression and thus affects cell proliferation and apoptosis. Foxf2 regulated the expression of Noxa, and siRNA-mediated depletion of Noxa prevented an increase in cell death induced by the loss of Foxf2 expression as assessed by quantitative RT-PCR. Figure S5. EGF ligand-mediated EGF receptor signaling overcomes Foxf2-controlled cell survival. Foxf represses the expression of EGF receptor ligands as assessed by quantitative RT-PCR. Supplementary material and methods. Detailed information is given on the antibodies and reagents, on biochemical and cell biological methods, and on RNA sequencing and bioinformatics analysis used in the study. Table S1. Excel file summarizing the differential expression analysis (siFoxf2 to siCtrl after 4 days TGFβ treatment or siCtrl with vs without TGFβ for 4 days) of all transcripts detected with RNA-sequencing. Table S2. Excel file showing the list of genes belonging to the different gene signatures (modules) and the strength of their modular membership (kME values). (ZIP 14675 kb) [file 13058_2018_1043_MOESM1_ESM.zip › Supplemental Information.pdf]

Foxf2 plays a dual role during TGFβ-induced EMT by promoting apoptosis yet enabling cell junction dissolution and migration

Nathalie Meyer-Schaller, Chantal Heck, Stefanie Tiede, Mahmut Yilmaz, and Gerhard Christofori

Additional file 1

Figures

Meyer-Schaller & Heck et al., Suppl. Figure S1

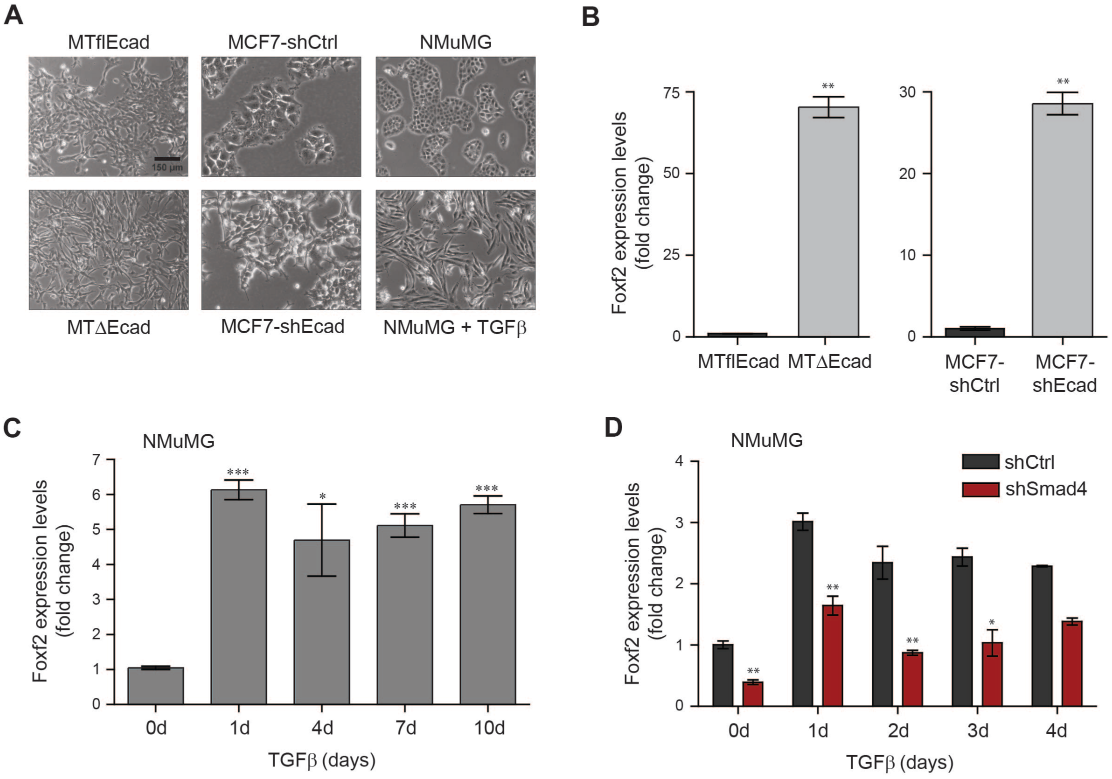

Figure S1. Foxf2 expression is upregulated during EMT via the canonical Smad pathway.

(A) MTflEcad, MCF7 and NMuMG cells undergo EMT upon deletion of the E-cadherin gene by expression of Cre-recombinase (MTΔEcad), shRNA expression against E-cadherin (MCF7-shEcad) or treatment with TGFβ (NMuMG/E9 + TGFβ), respectively. Representative phase contrast microphotographs are shown. Scale bar, 50μm.

(B-D) Foxf2 mRNA levels were determined by quantitative RT-PCR in MTflEcad/MTΔEcad and MCF7-shCtrl/MCF7-shEcad cells (B), in NMuMG cells treated with TGFβ for the indicated times (C) or in stable Smad4 knockdown (shSmad4) and control (shCtrl) NMuMG cells treated with TGFβ for the indicated times (D). Values were normalized to RPL19 and reported as fold changes to untreated shCtrl cells. Data are shown as mean ±SEM of two independent experiments. Statistical values were calculated by using a paired two-tailed t-test, either comparing to untreated control cells (B-C) or to shCtrl cells for each time point (D). *$p \leq 0.05$; **$p \leq 0.01$; ***$p \leq 0.001$.

Meyer-Schaller & Heck et al., Suppl. Figure S2

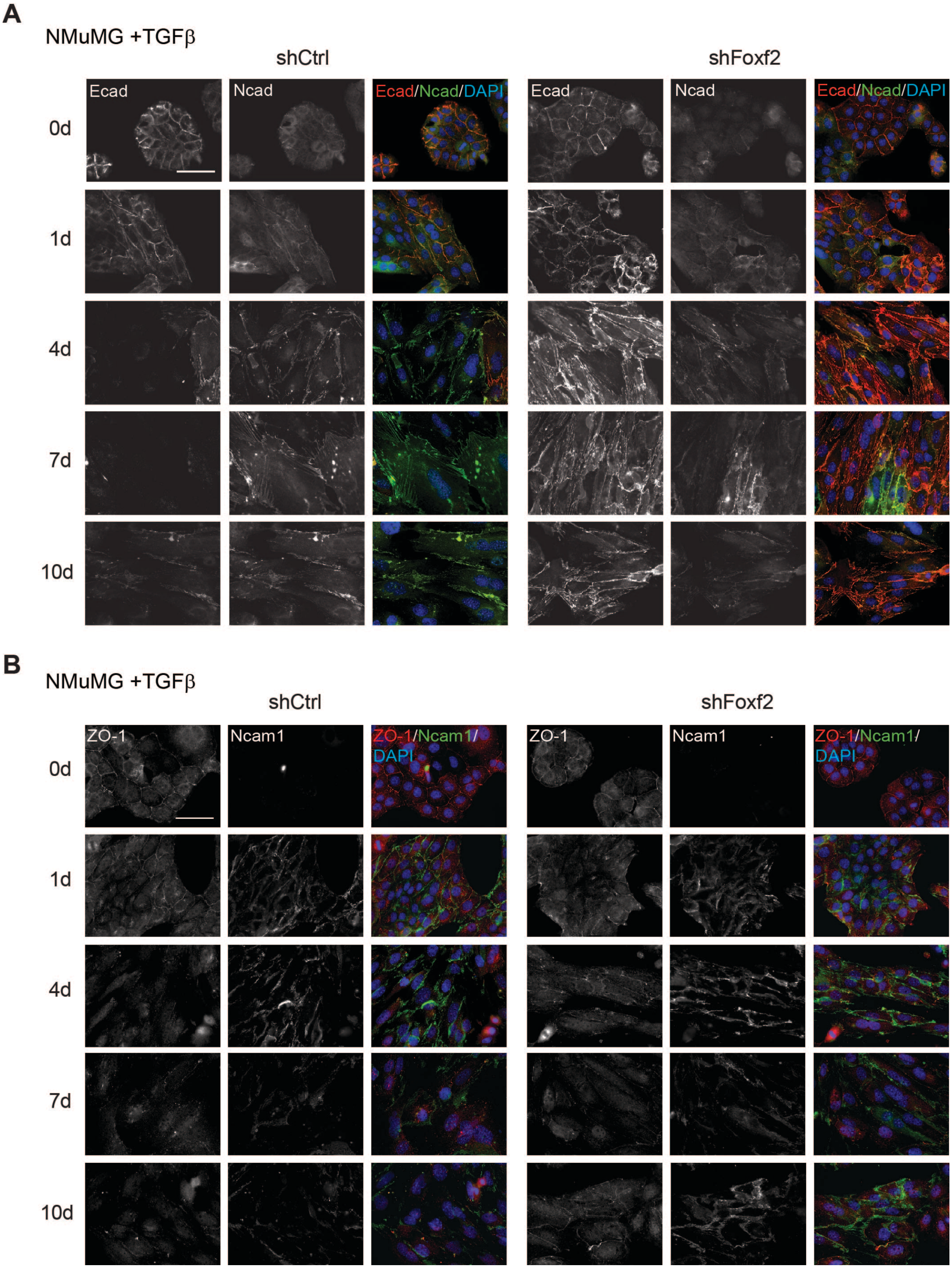

C

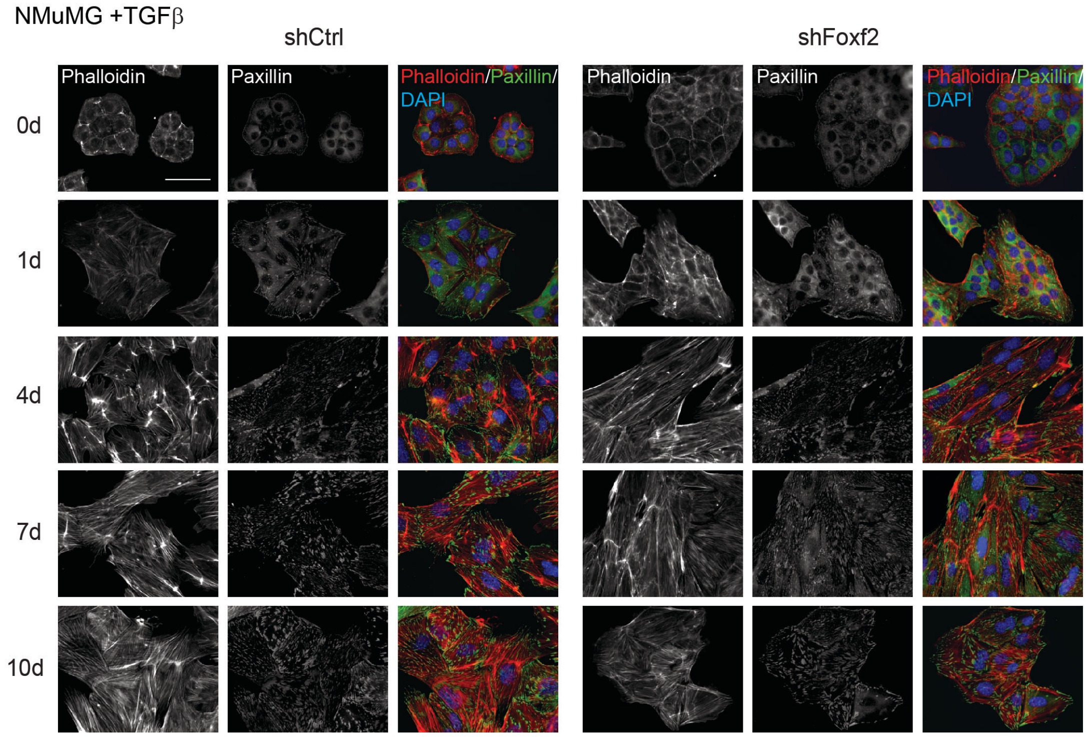

Figure S2. Foxf2 is required for TGFβ-induced disruption of adherens junctions. Representative immunofluorescence stainings of epithelial and mesenchymal marker proteins in shFoxf2 knockdown and shCtrl NMuMG cells treated with TGFβ for the times indicated using confocal microscopy.

(A) E-cadherin (red) and N-cadherin (green), **(B)** ZO-1 (red) and Ncam1 (green), **(C)** phalloidin (red; to stain filamentous actin) and paxillin (green; to stain focal adhesions).

Nuclei are visualized by DAPI staining (blue). Size bars, 50μm.

Meyer-Schaller & Heck et al., Suppl. Figure S3

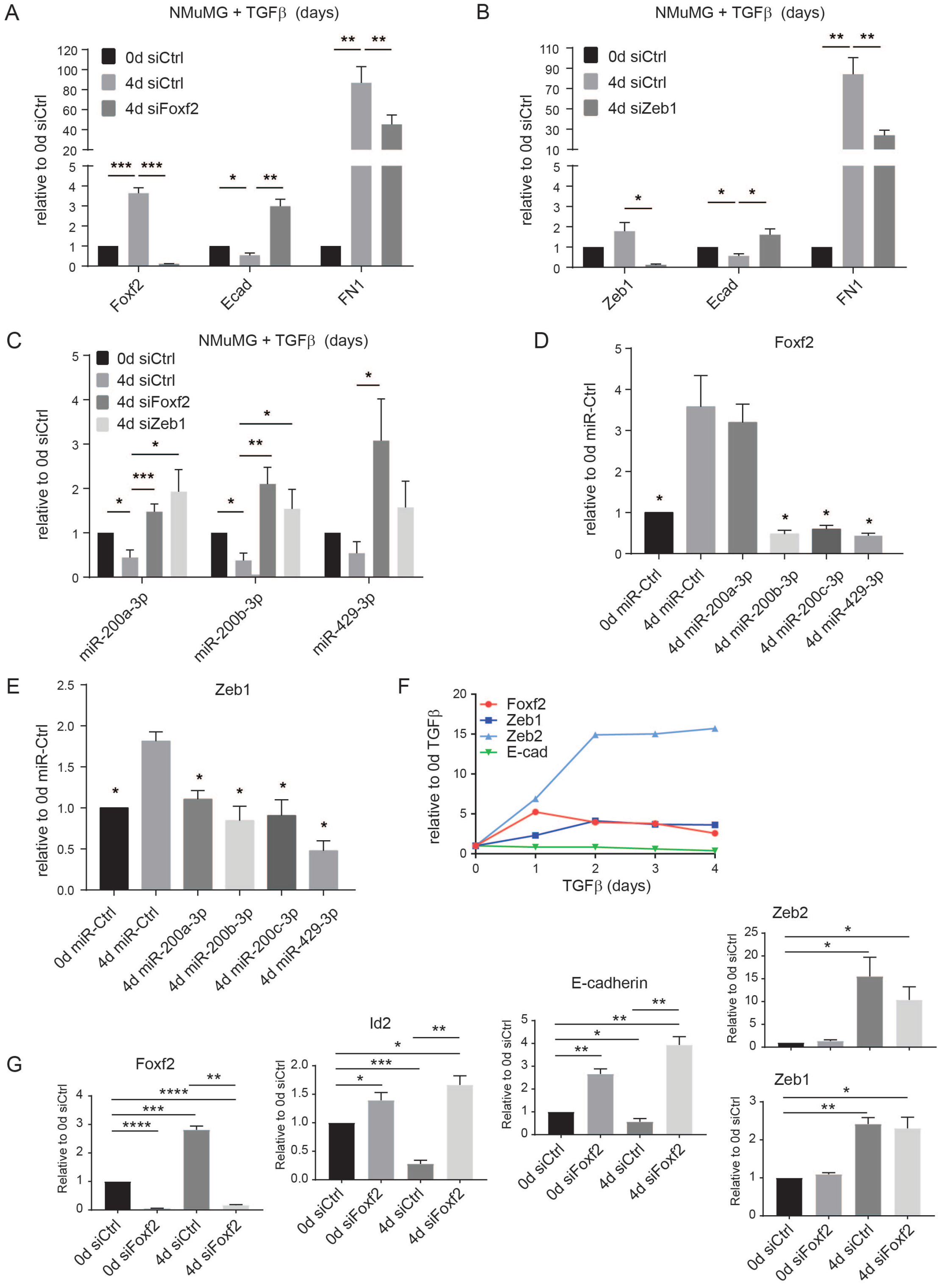

Figure S3. Foxf2 regulates the expression of Zeb1, Zeb2, Id2 and members of the miR-200 family.

(A) Quantitative RT-PCR was used to determine the expression of Foxf2, E-cadherin (Ecad) and fibronectin (FN1) in NMuMG cells treated with siControl (siCtrl) or siFoxf2 in the presence or absence of TGFβ for 4 days as indicated.

(B) Quantitative RT-PCR was used to determine the expression of Zeb1, Ecad, FN1 in NMuMG cells treated with siCtrl or siZeb1 in the presence or absence of TGFβ for 4 days as indicated.

(C) Quantitative RT-PCR was used to determine the expression of miR-200a-3p, miR-200b-3p and miR-429-3p in NMuMG cells treated with siCtrl, siZeb1 or siFoxf2 in the presence or absence of TGFβ for 4 days as indicated.

(D,E) Quantitative RT-PCR was used to determine the expression of Foxf2 (D) or Zeb1 (E) in NMuMG cells treated with miRNA control (miR-Ctrl) or miR-200a-3p, miR-200b-3p, miR-200c-3p and miR-429-3p in the presence or absence of TGFβ for 4 days as indicated. Statistical significant changes to 4d miR-Ctrl are indicated by asterisks.

(F) Quantitative RT-PCR was used to determine the expression of Foxf2, Zeb1, Zeb2 and Ecad in NMuMG cells treated with TGFβ for 1, 2, 3 and 4 days or left untreated (0d). Expression levels are shown as an example from one experiment and are normalized to the untreated control.

(G) Quantitative RT-PCR was used to determine the expression of Foxf2, Id2, Ecad, Zeb1 and Zeb2 in Py2T cells, derived from a tumor of the MMTV-PyMT mouse model of breast cancer, treated with siRNA control (siCtrl) or Foxf2 specific siRNA (siFoxf2) in the presence or absence of TGFβ for 4 days as indicated.

Values were normalized to RPL19 (A,B,D,E,F,G) or miR-U6 (C) and reported as fold changes to untreated siCtrl/miR-Ctrl cells. Data are shown as mean ±SEM of at least 3 independent experiments (except for F). Statistical values were calculated by using a paired/unpaired two-tailed t-test. *$p \leq 0.05$; **$p \leq 0.01$; ***$p \leq 0.001$.

Meyer-Schaller & Heck et al., Suppl. Figure S4

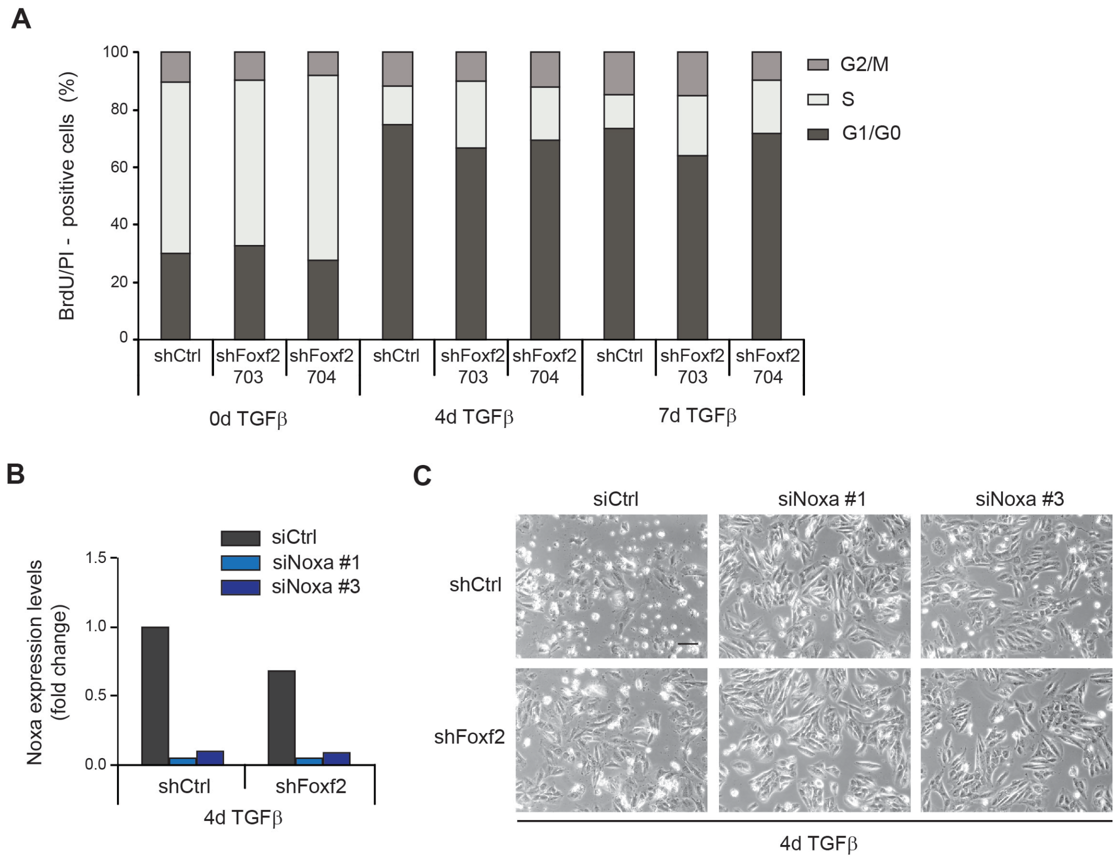

Figure S4. Foxf2 regulates Noxa expression and thus affects cell proliferation and apoptosis.

(A) Foxf2 downregulation leads to a minor increase in proliferation. shFoxf2 cells and shCtrl-expressing NMuMG cells were treated with TGFβ for the times indicated and proliferation was assessed by BrdU incorporation and PI staining followed by flow cytometry analysis.

(B) shFoxf2 and shCtrl-expressing NMuMG cells were transfected with control siRNA (siCtrl) and two different siRNAs specific for murine Noxa (siNoxa #1, siNoxa #3) and incubated with TGFβ for the indicated times. Noxa mRNA levels were determined by quantitative RT-PCR and values were normalized to RPL19 and reported as fold changes to untreated shCtrl cells.

(C) Phase contrast micrographs of shFoxf2 and shCtrl-expressing NMuMG cells transfected with siCtrl or siNoxa #1/3 and treated with TGFβ for 4 days. Scale bar, 100μm.

Meyer-Schaller & Heck et al., Suppl. Figure S5

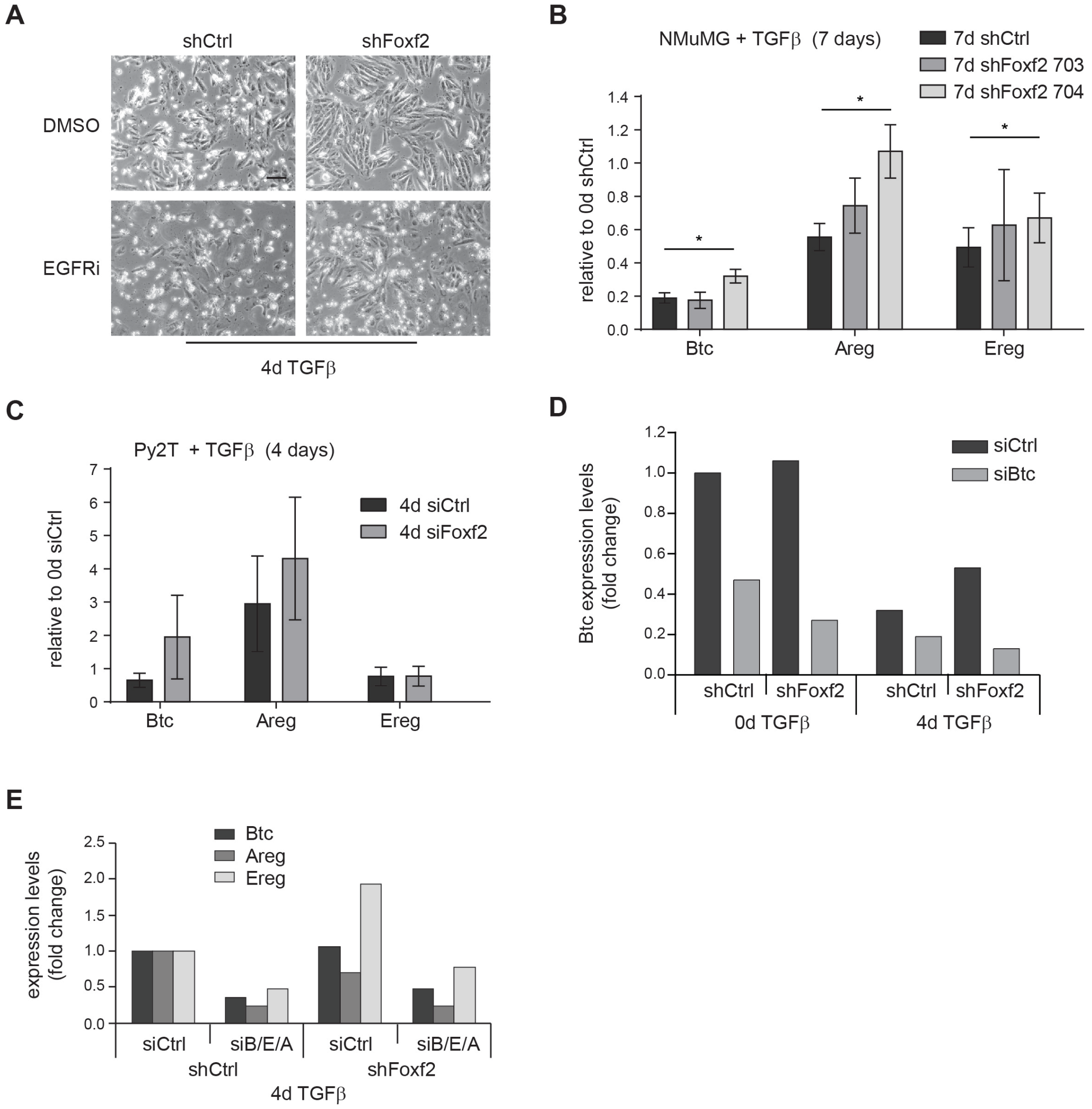

Figure S5. EGF ligand-mediated EGF receptor signaling overcomes Foxf2-controlled cell survival.

(A) Phase contrast micrographs of NMuMG cells expressing shRNA specific for Foxf2 (shFoxf2) or control shRNA (shCtrl) treated with EGFR inhibitor (EGFRi) or solvent control (DMSO) together with TGFβ for four days. Scale bar, 100µm.

(B,C) mRNA levels were determined by quantitative RT-PCR in shFoxf2 and shCtrl-expressing NMuMG cells (B) or in Py2T cells derived from a tumor of the MMTV-PyMT mouse model of breast cancer after siRNA-mediated knockdown of Foxf2 or siCtrl (C), treated with TGFβ for the indicated days. Values were normalized to RPL19 and reported as fold changes to untreated shCtrl/siCtrl cells. Data are shown

as mean ±SEM of three independent experiments. Statistical values were calculated between sh/siCtrl and sh/siFoxf2 in the presence of TGFβ, using a paired two-tailed t-test. *$p \leq 0.05$; betacellulin (Btc); amphiregulin (Areg); epiregulin (Ereg)

(D) Betacellulin (Btc) mRNA levels were determined by quantitative RT-PCR in shFoxf2 and shCtrl-expressing NMuMG cells depleted of Btc expression by transfection with a siRNAs against Btc or control siRNA (siCtrl) and treated with TGFβ for the indicated days. Values were normalized to RPL19 and reported as fold changes to untreated shCtrl&siCtrl cells.

(E) Betacellulin (Btc), amphiregulin (Areg) and epiregulin (Ereg) mRNA levels were determined by quantitative RT-PCR in shFoxf2 and shCtrl-expressing NMuMG cells depleted of Btc, Areg and Ereg expression by the combined transfection with siRNAs against Btc, Areg and Ereg or control siRNA (siCtrl) and treated with TGFβ for four days. Values were normalized to RPL19 and presented as fold changes to untreated shCtrl&siCtrl cells.

Table S1: See separate Excel File. Differential expression analysis (siFoxf2 to siCtrl after 4d TGFβ treatment or siCtrl with vs without TGFβ for 4d) of all transcripts detected with RNA-sequencing.

Table S2: See separate Excel File. List of genes belonging to the different gene signatures (modules) and the strength of their modular membership (kME values).

Supplementary Material and Methods

Reagents and Antibodies

Recombinant human TGFβ (#240-B, R&D Systems), small hairpin and silencing RNAs: shRNAs and siRNAs were purchased from Sigma-Aldrich, Control shRNA (shCtrl, Mission Non-Target shRNA control vector, #SHC002), murine Foxf2 shRNAs (MISSION® shRNA bacterial glycerol stock, shFoxf2 703 #TRCN0000084958, shFoxf2 704 #TRCN0000084960), murine betacellulin siRNA (MISSION® siRNA, siBtc, #SASI_Mm02_00311942), murine epiregulin siRNA

(MISSION® siRNA, siEreg, #SASI_Mm01_00072956), murine amphiregulin siRNA (MISSION® siRNA, siAreg, #SASI_Mm02_00316835), murine Noxa siRNA (MISSION® siRNA, siNoxa #1,3 #SASI_Mm01_00077286,8). siRNA for RNA-sequencing and miRNA were ordered from Ambion (ThermoFisher Scientific): murine Foxf2 siRNA (Silencer Select siRNA #s66122) with siCtrl (Silencer Select Negative Control No.2 siRNA #4390847), mmu-miR-200a-3p (#PM10991), mmu-miR-200b-3p (#PM10492), mmu-miR-200c-3p (#PM11714), mmu-miR-429-3p (#PM10759) with miCtrl (pre-miR miRNA precursor molecules negative control #1, AM17110). Antibodies: E-cadherin for immunofluorescence (#13-1900, Zymed), for immunoblotting (#610182, Transduction Laboratories), ZO-1 (#617300, Zymed), paxillin (#P13520, Transduction Laboratories), fibronectin (#F-3648, Sigma-Aldrich), N-CAM (#9672, Sigma), N-cadherin for immunofluorescence (#610921, Transduction Laboratories), for immunoblotting (#33-3900, Zymed), PARP (#9542, Cell Signaling), EGFR (#2232, Cell Signaling), pEGFR (Y1173) (#sc-12351, Santa Cruz), cleaved Caspase-3 (#9664, Cell Signaling), tubulin (#T-9026, Sigma-Aldrich), actin (#sc-1616, Santa Cruz), HA-tag for ChIP (#3724, Cell signaling) or IgG from rabbit serum as ChIP control (#I-5006, Sigma). Alexa Fluor 568-coupled phalloidin from ThermoFisher Scientific (A12380) was used to visualize filamentous actin. Inhibitors: EGFR inhibitor AG1478 (#ALX-270-036, Alexis Biochemicals).

Primers for quantitative RT-PCR

murine RPL19: fwd: 5'-CTCGTTGCCGGAAAAACA-3', rev:5'-TCATCCAGGTCACCTTCTCA-3'; human RPL19: fwd: 5'-GATGCCGGAAAAACACCTTG-3'; rev: 5'-CAGGGCAGTGATCTCCTTCTG-3'; murine Foxf2: fwd: 5'-AGCAGAGCTACTTGCACCAGA-3', rev: 5'-GCAGTCCGACTGAGAGATCCT-3'; human Foxf2: fwd: 5'-AGCAGAGCTACTTGCACCAGA-3', rev: 5'-GCAGTCCCACTGAGAGGTCCT-3'; murine E-cadherin: fwd: 5'-CGACCCTGCCTCTGAATCC-3', rev: 5'-TACACGCTGGGAAACATGAGC-3'; murine Zeb1: fwd: 5'-GCCAGCAGTCATGATGAAAA-3', rev: 5'-TATCACAATACGGGCAGGTG-3'; murine Zeb2: fwd: 5'-GGAGGAAAAACGTGGTGAACTAT-3', rev: 5'-GCAATGTGAAGCTTGTCCTCTT-3'; murine Id2: fwd: 5'-ACTATCGTCAGCCTGCATCA-3', rev: 5'-AGCTCAGAAGGGAATTCAGATG-

3'; murine Noxa: fwd: 5'-CAGATGCCTGGGAAGTCG-3', rev: 5'-TGAGCACACTCGTCCTTCAA-3'; murine Btc: fwd: 5'-ACCAATGGCTCTCTTTGTGG-3', rev: 5'-CCGAGAGAAGTGGGTTTTCA-3'; murine Ereg: fwd: 5'-TTGACGCTGCTTTGTCTAGG-3', rev: 5'-GGATCACGGTTGTGCTGAT-3'; murine Areg: fwd: 5'-AAGAAAACGGGACTGTGCAT-3', rev: 5'-GGCTTGGCAATGATTCAACT-3'; murine miR-U6 fwd: TGGCCCCTGCGCAAGGATG; murine miR-200a-3p fwd: TAACACTGTCTGGTAACGATG; murine miR-200b-3p fwd: TAATACTGCCTGGTAATGATG; murine miR-429-3p fwd: TAATACTGTCTGGTAAGCCGT; for all miRNA universal reverse primer from QuantiMir RT kit (BioCat) was used.

Primers for ChIP

Intergenic primer: fwd: 5'-GCTCCGGGTCCTATTCTTGT-3', rev: 5'-TCTTGGTTTCCAGGAGATGC-3'; murine Btc (-450 to -253): fwd: 5'-CTGCGTCAACTGTCAAATGC-3', rev: 5'-AAGAGGACCTGGTCATGTGG-3'; murine Ereg (-851 to -654): fwd: 5'-GCATTTGAGACAGGCACAGA-3', rev: 5'-CCCTCAGCTTCCAATGTGAT-3'; murine Areg (exon2 +1086 to 1210): fwd: 5'-CATTATGCAGCTGCTTTGGA-3', rev: 5'-TTTCGCTTATGGTGGAAACC-3'; murine Noxa (-696 to -499): fwd: 5'- TTCCTCCACAAAGGATCTGG-3', rev: 5'-TGCGTAGTGACTTGCGACTT-3'.

Immunoblotting

Cells were lysed for 20min on ice in RIPA-buffer (150mM NaCl, 2mM MgCl, 2mM $CaCl_2$, 0.5% NaDOC, 1% NP40, 0.1% SDS, 10% Glycerol, 50mM Tris pH8.0, 2mM Na_3VO_4, 10mM NaF, 1mM DTT, and a 1:200 dilution of stock protease inhibitor cocktail for mammalian cells (Roche). Protein concentration was determined using DC™ Protein Assay (BioRad Laboratories). Equal amounts of protein were diluted in SDS-PAGE loading buffer (10% glycerol, 2% SDS, 65mM Tris, 1mg/100ml Bromphenolblue, 1 % β-mercaptoethanol) and resolved by SDS-PAGE. SDS-PAGE gels were transferred to polyvinylidene fluoride (PVDF) membranes (Millipore) by semi-dry transfer, blocked with 5% skim milk powder in Tris-buffered saline with

0.05% Tween 20 (TBST) and incubated with the indicated antibodies. HRP conjugated antibodies were detected using enhanced chemiluminescence.

Immunofluorescence staining

shCtrl or shFoxf2 NMuMG cells were plated on glass coverslips and treated for the indicated times with TGFβ (2ng/ml). Cells were fixed using 4% paraformaldehyde /PBS for 10min and permeabilized with 0.2% Triton X100 for 2min at room temperature. Then cells were blocked using 3% BSA, 0.01% TritonX100 in PBS for 1h at room temperature, incubated with the indicated primary antibody for 1h followed by incubation with the fluorochrome-labelled secondary antibody (Alexa Fluor®, Invitrogen) for 1h at room temperature. Nuclei were stained with 6-diamidino-2-phenylindole (DAPI; 1µg/ml) (Sigma-Aldrich) for 10min. The coverslips were mounted (Fluorescent mounting medium, Dako) on microscope slides and imaged using a Leica SP5 confocal microscope.

RNA-sequencing and data analysis

For RNA-sequencing analysis, NMuMG cells were transfected with siRNA control (siCtrl) or siFoxf2 two days prior to TGFβ addition and subsequently retransfected three days after the first transfection. Cells were incubated for 4 days with TGFβ or left untreated (epithelial control). RNA was isolated using miRNeasy kit (Qiagen) and 200ng of RNA was used to generate sequencing libraries (TruSeq Stranded mRNA LT Sample Prep Kit). Sequencing was performed on a HiSeq 2500 machine using HiSeq SBS Kit v4.

Single-end RNA-sequencing reads (51-mers) were mapped to the mouse genome assembly version mm10 using RNA-STAR [1] (default parameters except for allowing only unique hits to genome (outFilterMultimapNmax=1) and filtering reads without evidence in spliced junction table (outFilterType="BySJout"). Using qCount function from the QuasR package [2] (version 3.12.1) together with the RefSeq mRNA coordinates from UCSC (genome.ucsc.edu, downloaded in Dec 2015), we counted the reads per gene that started in any annotated exon of the gene. Differential expression was calculated using DE-Seq2 [3] (DESeq2_1.12.4) including the experiment batch and conditions in the multifactorial design. Genes with adjusted p-

value smaller than 0.05 and at least absolute log2 fold change of 1 were considered to be differentially expressed.

To extract gene signatures, weighted gene coexpression analysis was performed with the R package WGCNA [4, 5] (R version 3.3.0, WGCNA_1.51). As input the 10,000 most variant genes were used based on their rlog values (calculated with DE-Seq2, multifactorial design including batch and condition, blind=TRUE, and batch effects removed with the removebatcheffect function from the limma package [6] (limma_3.30.6)). Modules (gene signatures) were generated using biweight midcorrelation, signed network type with minimal module size of 300. The eigengene expression was plotted across samples using a barplot while the expression of the individual genes per module (gene signature) (scaled rlog values) was visualized in a heatmap with samples clustered by the default hclust function using gplots (gplots_3.0.1). Genes belonging to the brown or yellow gene signature were further analysed for pathway enrichment using IPA (Ingenuity Pathway Analysis).
The RNA expression data from the RNA-Sequencing are deposited at Gene Expression Omnibus (GEO accession number: GSE112796).

Survival and metastasis correlation analysis and tumor expression analysis of Foxf2
Analyses of Foxf2 expression correlation with breast cancer patient survival or metastasis formation were performed using various distinct datasets of the Netherlands Cancer Institute (NKI295) [7], of the Memorial Sloan-Kettering Cancer Center (MSKCC) NY [8] and the Metabric consortium [9, 10]. The NKI295 database contained 288 tumors of early-stage breast cancer (stageI and stageII) with information on Foxf2 expression, overall survival (10y) and time to metastasis. Tumors were divided into two groups based on the relative expression of Foxf2 to the tumor pools (logFC = 0) and further stratified by ER status (N=68 ER-, N=220 ER+). The Minn database consisted of microarray expression data from 82 patients with more advanced (T2-T4) mammary carcinomas and clinical information on distant metastasis free survival. Tumors were divided into two groups based on the median expression of Foxf2 and further stratified by LN status (N=54 LN+, N=28 LN-). The Metabric database contains 1296 patients with complete clinical and transcriptomics information of which 352 belong to the luminal subtype B. Tumors were divided into two groups based on the median expression of Foxf2.

With all datasets, overall or metastasis-free survival was estimated by applying Kaplan-Meier survival analysis and Cox proportional hazards regression modeling with the R survival package 2.36-5 and R version 2.11.1 (www.r-project.org). The p-value of the likelihood-ratio test was used to assess the statistical significance between the different patient groups.

To compare the expression levels of Foxf2 across different tumor subtypes, available clinical data was used to define ER, PR, HER2 and TN (triple negative ER-PR-HER2-) status. The classification of claudin-low subtype tumors was performed as described by Prat et al. [11]. Statistical significance of expression differences between a specific tumor subtype and all other tumors was assessed using the Kruskal-Wallis test.

References

1. Dobin A, Davis CA, Schlesinger F, Drenkow J, Zaleski C, Jha S, Batut P, Chaisson M, Gingeras TR: **STAR: ultrafast universal RNA-seq aligner**. *Bioinformatics* 2013, **29**(1):15-21.
2. Gaidatzis D, Lerch A, Hahne F, Stadler MB: **QuasR: quantification and annotation of short reads in R**. *Bioinformatics* 2015, **31**(7):1130-1132.
3. Love MI, Huber W, Anders S: **Moderated estimation of fold change and dispersion for RNA-seq data with DESeq2**. *Genome Biol* 2014, **15**(12):550.
4. Langfelder P, Horvath S: **WGCNA: an R package for weighted correlation network analysis**. *BMC Bioinformatics* 2008, **9**:559.
5. Langfelder P, Horvath S: **Fast R Functions for Robust Correlations and Hierarchical Clustering**. *J Stat Softw* 2012, **46**(11).
6. Ritchie ME, Phipson B, Wu D, Hu Y, Law CW, Shi W, Smyth GK: **limma powers differential expression analyses for RNA-sequencing and microarray studies**. *Nucleic Acids Res* 2015, **43**(7):e47.
7. van 't Veer LJ, Dai H, van de Vijver MJ, He YD, Hart AA, Mao M, Peterse HL, van der Kooy K, Marton MJ, Witteveen AT *et al*: **Gene expression profiling predicts clinical outcome of breast cancer**. *Nature* 2002, **415**(6871):530-536.
8. Minn A, Gupta G, Siegel P, Bos P, Shu W, Giri D, Viale A, Olshen A, Gerald W, Massague J: **Genes that mediate breast cancer metastasis to lung.** *Nature* 2005, **436**(7050):518-524.
9. Curtis C, Shah SP, Chin SF, Turashvili G, Rueda OM, Dunning MJ, Speed D, Lynch AG, Samarajiwa S, Yuan Y *et al*: **The genomic and transcriptomic architecture of 2,000 breast tumours reveals novel subgroups**. *Nature* 2012, **486**(7403):346-352.
10. Dvinge H, Git A, Graf S, Salmon-Divon M, Curtis C, Sottoriva A, Zhao Y, Hirst M, Armisen J, Miska EA *et al*: **The shaping and functional consequences of the microRNA landscape in breast cancer**. *Nature* 2013, **497**(7449):378-382.

11. Prat A, Parker JS, Karginova O, Fan C, Livasy C, Herschkowitz JI, He X, Perou CM: **Phenotypic and molecular characterization of the claudin-low intrinsic subtype of breast cancer**. *Breast Cancer Res* 2010, **12**(5):R68.
